# Spectroscopic Signatures of Structural Disorder and Electron‐Phonon Interactions in Trigonal Selenium Thin Films for Solar Energy Harvesting

**DOI:** 10.1002/smtd.202501841

**Published:** 2026-01-14

**Authors:** Rasmus S. Nielsen, Axel G. Medaille, Arnau Torrens, Oriol Segura‐Blanch, Seán R. Kavanagh, David O. Scanlon, Aron Walsh, Edgardo Saucedo, Marcel Placidi, Mirjana Dimitrievska

**Affiliations:** ^1^ Nanomaterials Spectroscopy and Imaging, Transport at Nanoscale Interfaces Laboratory Swiss Federal Laboratories for Material Science and Technology (EMPA) Duebendorf Switzerland; ^2^ Universitat Politècnica de Catalunya (UPC) Photovoltaic Lab ‐ Micro and Nano Technologies Group (MNT) Electronic Engineering Department, EEBE Barcelona Spain; ^3^ Universitat Politècnica de Catalunya (UPC), Barcelona Centre for Multiscale Science & Engineering Barcelona Spain; ^4^ Harvard University Center for the Environment Cambridge Massachusetts USA; ^5^ School of Chemistry University of Birmingham Birmingham UK; ^6^ Thomas Young Centre and Department of Materials Imperial College London London UK; ^7^ Department of Physics Ewha Womans University Seoul South Korea

**Keywords:** electron‐phonon coupling, optoelectronics, photoluminescence, Raman, selenium

## Abstract

Selenium is experiencing renewed interest as a elemental semiconductor for a range of optoelectronic and energy applications due to its irresistibly simple composition and favorable wide bandgap. However, its high volatility and low radiative efficiency make it challenging to assess structural and optoelectronic quality, calling for advanced, non‐destructive characterization methods. In this work, we employ a closed‐space encapsulation strategy to prevent degradation during measurement and enable sensitive probing of vibrational and optoelectronic properties. Using temperature‐dependent Raman and photoluminescence spectroscopy, we investigate grown‐in stress, vibrational dynamics, and electron‐phonon interactions in selenium thin films synthesized under nominally identical conditions across different laboratories. Our results reveal that short‐range structural disorder is not intrinsic to the material, but highly sensitive to subtle processing variations, which strongly influence electron‐phonon coupling and non‐radiative recombination. We find that such structural disorder and grown‐in stress likely promote the formation of extended defects, which act as dominant non‐radiative recombination centers limiting carrier lifetime and open‐circuit voltage in photovoltaic devices. These findings demonstrate that the optoelectronic quality of selenium thin films can be significantly improved through precise control of synthesis and post‐deposition treatments, outlining a clear pathway toward optimizing selenium‐based thin film technologies through targeted control of crystallization dynamics and microstructural disorder.

## Graphical abstract

Selenium plays hard to get ‐ volatile, low radiative efficiency, and highly sensitive to processing. Using a closed‐space encapsulation strategy, we probe room‐temperature photoluminescence and quantify structural disorder, showing how precise structural control can turn selenium into a high‐performance optoelectronic material of tomorrow.

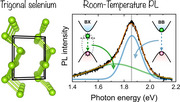

## 1 Introduction

**FIGURE 1:**
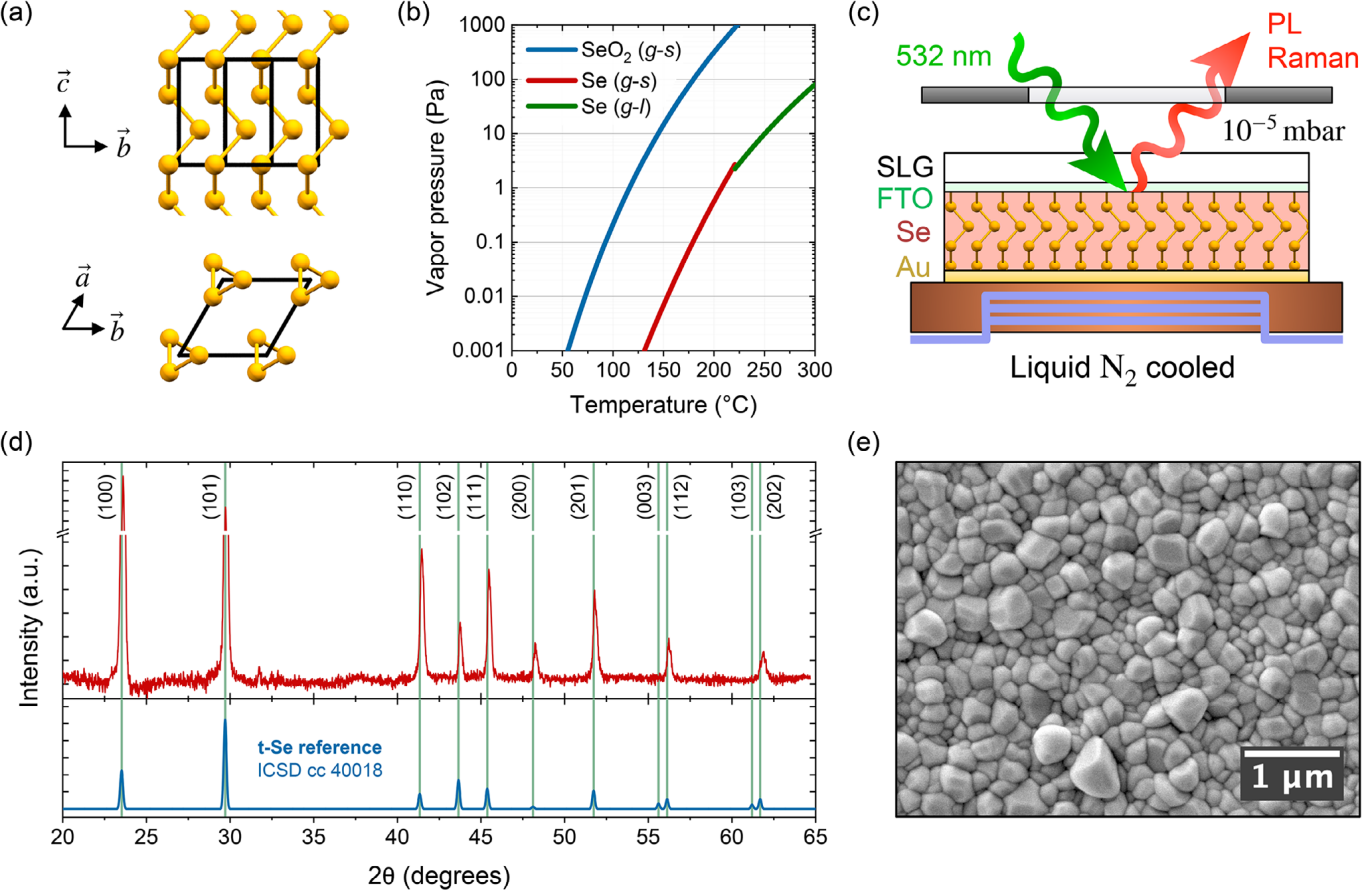
(a) Crystal structure of trigonal selenium viewed along different lattice directions. (b) Vapor pressure of selenium and selenium dioxide as a function of temperature; *g*, *l*, and *s* denote gas, liquid, and solid phases, respectively. Data from Xu et al. ^[^ ^41^ ^]^ (c) Device schematic of the closed‐space encapsulated selenium thin film in an optical cryostat cooled using liquid nitrogen at ∼10^−5^ mbar. (d) XRD pattern of the polycrystalline selenium thin film, shown above the reference powder pattern for trigonal selenium (ICSD collection code 40018); all observed reflections match the reference. (e) Top‐view SEM image of the polycrystalline selenium thin film.

Selenium is a wide‐bandgap elemental semiconductor that has attracted renewed interest for a range of optoelectronic applications, including photodetectors [1, 2, 3], radiation sensors [4, 5], and, most notably, indoor [6, 7, 8, 9, 10, 11] and tandem photovoltaics [12, 13, 14, 15, 16]. Its quasi‐1D trigonal crystal structure, shown in Figure 1a, gives rise to pronounced anisotropy in material properties, enabling more advanced technologies such as polarization‐sensitive light sources and detectors [17, 18, 19, 20]. In addition to its low‐dimensional structure, trigonal selenium offers a direct bandgap in the 1.8‐2.2 eV range [21, 22, 23], which is tunable through alloying with tellurium [24, 25, 26], a high optical absorption coefficient [6, 27], tolerance to intrinsic point defects [28, 29, 30], low‐temperature processing [31, 32, 33, 34], long‐term air stability [35, 36], and the irresistible simplicity of a single‐element system. Yet despite these advantages, the optoelectronic quality of selenium thin films remains a major bottleneck, limiting progress toward high‐efficiency photovoltaic devices [27, 28, 37]. Photoluminescence (PL) and Raman spectroscopy is widely recognized as a powerful non‐contact characterization tools for evaluating optoelectronic and structural quality of materials [38, 39, 40]. Therefore, a systematic investigation of the radiative emission properties and microstructural quality using these techniques is a natural next step toward identifying both intrinsic and synthesis‐induced performance limitations in state‐of‐the‐art selenium thin films.

PL studies of trigonal selenium date back to the 1960s and 1970s, but focus exclusively on bulk crystals grown via vapor phase or melt growth and measured at cryogenic temperatures. Queisser et al. reported polarization‐dependent PL from selenium crystals submerged in liquid hydrogen, revealing sharp spectral lines attributed to bound excitons [42]. Zetsche et al. observed numerous phonon satellites in PL spectra collected between 2 and 50 K [43], and Kuhler et al. performed electro‐PL measurements under applied electric fields to probe impurity‐related transitions [44]. Later, Moreth identified sharp emission lines from indirect free‐exciton decay at 1.4 K, enabling an unambiguous determination of the quasi‐indirect nature of the bandgap [45].

In contrast to free‐standing single crystals, selenium thin films are typically grown directly on substrates and are thus more susceptible to substrate‐induced stress, anisotropic grain growth, and structural disorder – all of which can significantly alter PL behavior. In 2022, Nielsen et al. reported two distinct PL emission bands at cryogenic temperatures from a selenium thin film integrated into a record‐performing photovoltaic device [27]. Liu et al. observed similar spectral features, although the acquisition conditions were not specified [46]. Other studies have reported redshifted PL peak energies well below the optical bandgap in films infiltrated into mesoporous oxide scaffolds [26, 47]. Li et al. presented PL spectra with peak energies more reasonable for the optical bandgap, but without a detailed analysis of the nature of the underlying transition [1]. This inconsistency across the literature underscores the need for a more systematic investigation. However, the high vapor pressure of selenium makes thin films prone to sublimation and degradation during PL measurements, even under low excitation conditions. This challenge may have limited the widespread use of PL as a diagnostic tool, despite its value for evaluating optoelectronic quality.

In this work, we use temperature‐dependent PL and Raman spectroscopy to study the optoelectronic and vibrational properties of selenium thin films synthesized in different laboratories using state‐of‐the‐art fabrication methodologies. To address the challenge posed by the high vapor pressure and volatility of selenium, we adopt a recently developed closed‐space encapsulation strategy introduced by Nielsen et al. [48], which effectively suppresses sublimation during the measurements. The vibrational properties are studied through a detailed analysis of the thermal evolution of Raman peaks, supported by first‐principles phonon dispersion calculations that enable identification of all Raman‐active modes and their associated atomic displacements. Temperature‐dependent PL measurements, complemented by power‐dependent excitation studies, are used to examine the nature of two emission bands and quantify the electron‐phonon interactions that drive the thermal shifts and broadening of the observed radiative features. By comparing samples produced in different labs, we show that the short‐range electron‐phonon coupling strength is highly sensitive to subtle processing variations. This demonstrates that a reduction of microstructural disorder through careful control of growth parameters and thermal processing can substantially improve the optoelectronic quality of selenium thin films.

## 2 Results

While the high vapor pressure and low melting point of selenium facilitate low‐cost fabrication, these properties complicate many standard characterization techniques, as the material readily sublimates under moderate heating and vacuum conditions. This challenge is intensified by surface oxidation, as selenium dioxide is a far more volatile species with an even higher vapor pressure, as shown in Figure 1b. To overcome these challenges, we adapted a closed‐space encapsulation strategy from Nielsen et al. [48], depositing a thin, chemically inert film on the absorber. This overlayer effectively prevents surface oxidation and sublimation, enabling vacuum measurements and significantly expanding the accessible temperature window without compromising the structural integrity of the selenium thin film. Optical access was preserved by collecting photoluminescence and Raman signals through the transparent substrate using a microscope objective, enabling higher‐sensitivity measurements than free‐space optics setups.

Using this encapsulation approach, we synthesized trigonal selenium thin films following a standard process flow established for state‐of‐the‐art devices developed by multiple research groups [12, 49, 50]; details are provided in the Methods section. The X‐ray diffraction (XRD) pattern in Figure 1d confirms the polycrystalline trigonal selenium phase, with all reflections matching the ICSD reference pattern (collection code 40018) and no strong crystallographic texture observed. The top‐view scanning electron microscopy (SEM), presented in Figure 1e, shows the polycrystalline selenium film with grain sizes consistent with previous reports. Following the structural characterization of the selenium thin film, the device structure illustrated in Figure 1c is completed by thermally evaporating a thin gold overlayer, enabling temperature‐ and power‐dependent Raman and photoluminescence spectroscopy.

### 2.1 Raman Spectroscopy

To interpret the vibrational properties of the trigonal selenium thin film, we first apply group theory to identify the expected phonon modes. Trigonal selenium crystallizes in space group *P*3
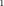
21 (No. 152), point group 32. Group theory predicts six phonon modes with the following irreducible representation [51]:

(1) $$\Gamma_{\mathrm{vib}}=A_{1}+2A_{2}+3E$$

where (i) $A_{2}+E$ are acoustic modes, (ii) $A_{2}+2E$ are infrared‐active modes, and (iii) $A_{1}+2E$ are Raman‐active modes. Here, A and E denote non‐degenerate and doubly degenerate modes, respectively, with respect to rotations about the principal crystallographic axis. The E modes in trigonal selenium are sometimes labeled with numerical subscripts (e.g., $E_{1}$, $E_{2}$), but since the crystal structure is non‐centrosymmetric and lacks inversion symmetry, this labeling is not appropriate. To avoid confusion with inversion parity classification, we instead label the two Raman‐active E modes as E(1) and E(2).

**FIGURE 2:**
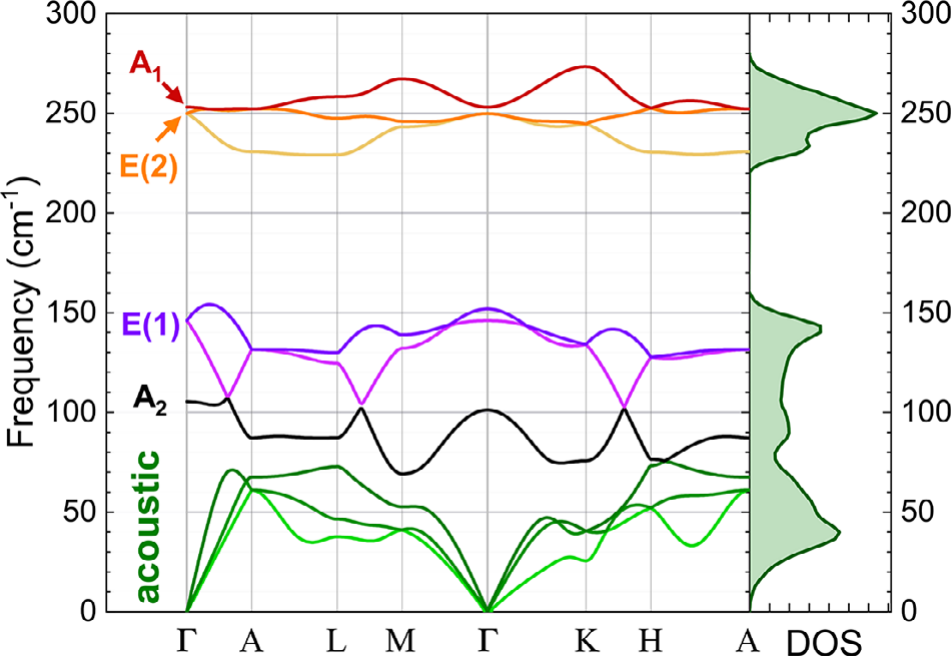
DFT‐calculated phonon dispersion along high‐symmetry directions, alongside the total phonon density of states (DOS). Acoustic and optical modes are labeled at the Γ‐point.

Building on this group‐theoretical framework, we next examine the phonon dispersion using first‐principles density functional theory (DFT) calculations. Figure 2 shows the phonon dispersion and the total phonon density of states (DOS), with vibrational modes at the Γ‐point labeled by the symmetries introduced above. The $A_{1}$ and doubly degenerate E(2) modes appear nearly degenerate in frequency at Γ, suggesting they may be difficult to distinguish experimentally. The spectrum also reveals a pronounced phonon bandgap separating the low‐ and high‐frequency optical branches. Such a gap substantially restricts the phase space for multi‐phonon scattering, which can suppress certain anharmonic decay pathways and, in some systems, enhance radiative efficiency by limiting rapid phonon‐mediated carrier thermalization [52, 53]. At the same time, concentrating the optical phonon density of states into a narrow set of high‐energy bond‐stretching modes means that photo‐excited carriers couple predominantly to these vibrations, which can strengthen electron–phonon interactions and promote non‐radiative recombination at extended defects [54, 55]. This dual role of the phonon bandgap, which both suppresses phonon‐phonon scattering and concentrates electron‐phonon coupling into a small number of high‐energy modes, is consistent with recent theoretical treatments showing that recombination rates are governed by the local vibrational modes available near defects [56]. Engineering the phonon dispersion – through alloying, nanostructuring, or controlled strain – may therefore offer pathways to tailor these competing effects and improve the optoelectronic performance potential.

**FIGURE 3:**
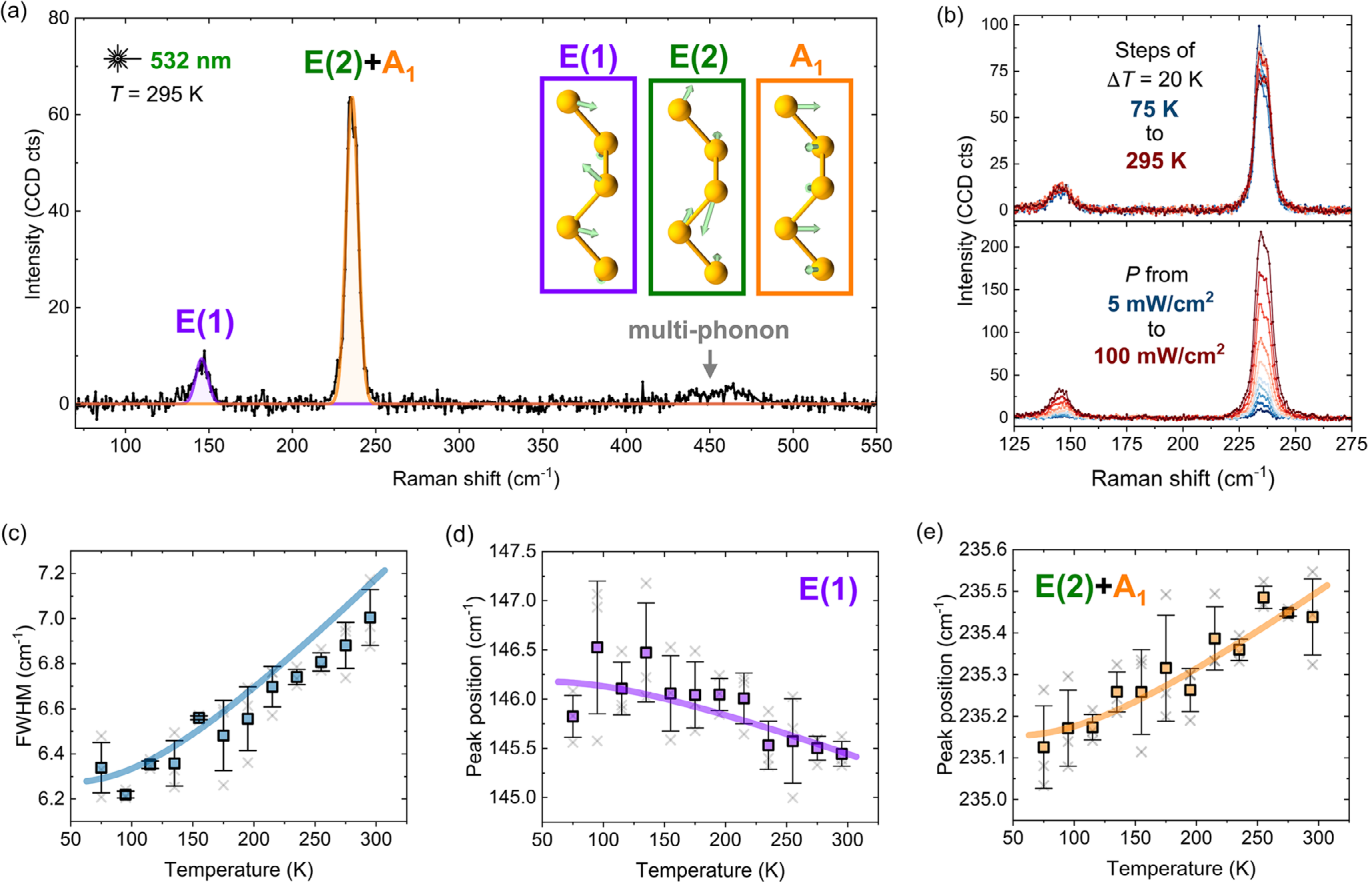
(a) Deconvoluted Raman spectrum showing the E(1) and E(2)+$A_{1}$ modes, with the latter modeled as a single peak due to their close proximity in frequency. The insets visualize the phonon displacement patterns for all three modes. (b) Raman spectra as a function of temperature and excitation power. (c) Full width at half maximum (FWHM) of Raman peaks versus temperature, constrained to be equal for both peaks. (d) Temperature dependence of the E(1) peak position. (e) Temperature dependence of the E(2)+$A_{1}$ peak position. The solid lines correspond to fitted curves. The results shown in panels (c–e) are averaged from three independent temperature‐dependent measurements; error bars represent one standard deviation.

Next, we deconvolute the experimental Raman spectrum acquired at room temperature using a rigorous methodology as detailed in Refs [57, 58], shown in Figure 3a. The low‐frequency peak at ∼146 ${\mathrm{cm}}^{-1}$ corresponds well with the doubly degenerate E(1) mode predicted by the phonon dispersion. The peak at ∼235 ${\mathrm{cm}}^{-1}$ overlaps with both the predicted E(2) and $A_{1}$ modes, which are nearly degenerate and indistinguishable in energy. The slight asymmetry of this peak, particularly evident in the power‐dependent Raman spectra in Figure 3b, suggests contributions from two distinct but overlapping vibrational modes. However, due to the finite linewidth, limited spectral resolution, and low signal intensity, the two modes are best modeled using a single Lorentzian. These assignments are consistent with prior observations by Geick et al. [59]. Additionally, a broad multi‐phonon band is observed between 430 and 475 ${\mathrm{cm}}^{-1}$, consistent with multi‐phonon scattering involving 2$A_{1}$, 2E(2) and $A_{1}$ + E(2) modes. The DFT‐calculated phonon displacements of the three Raman‐active modes are shown as an inset in Figure 3a, demonstrating that the E(1) mode is a bending type vibration that shears the covalent bonds, whereas both E(2) and $A_{1}$ correspond to asymmetric and symmetric bond‐stretching vibrations, respectively. To better visualize these displacements, additional representations along different lattice directions are provided in Figure S1 (Supporting Information).

Figure 3b presents the temperature‐ and power‐dependent Raman spectra. In addition to verifying the asymmetry of the ∼235 ${\mathrm{cm}}^{-1}$ peak, which supports the interpretation of overlapping E(2) and $A_{1}$ modes, the power‐dependent measurements are used to determine the maximum excitation power density and acquisition parameters that do not induce irreversible structural changes to the selenium thin film, thereby ensuring the integrity of subsequent Raman and photoluminescence measurements. The temperature‐dependent Raman spectra are deconvoluted using the same procedure as the room‐temperature spectrum in Figure 3a, with the E(2) and $A_{1}$ modes modeled as a single Lorentzian. The full width at half maximum (FWHM) is constrained to be equal for both fitted peaks, implying similar phonon lifetimes for all single‐phonon modes. The extracted FWHM and the two peak positions as a function of temperature are shown in Figure 3c–e, respectively.

To quantify the temperature dependence of the Raman peak positions and width, we fit the data using simple Bose‐Einstein models, which attribute both the peak shifts and broadening to anharmonic phonon‐phonon interactions. The peak positions are modeled as:

(2) $$E\left(T\right)=E_{0}-\frac{\lambda }{\exp\left(\hbar \bar{\omega }/k_{B}T\right)-1}$$

where $E_{0}$ is the mode energy at 0 K, λ is the anharmonic coupling constant, $\hbar \bar{\omega }$ is the effective phonon energy, and $k_{B}T$ is the thermal energy. The linewidth broadening is similarly modeled as:

(3) $$\Gamma \left(T\right)=\Gamma_{0}+\frac{\Gamma_{\mathrm{ph}}}{\exp\left(\hbar \omega_{\mathrm{ph}}/k_{B}T\right)-1}$$

where $\Gamma_{0}$ is the temperature‐independent broadening, $\Gamma_{\mathrm{ph}}$ is the phonon‐phonon coupling strength, and $\hbar \omega_{\mathrm{ph}}$ is the effective phonon energy. The solid lines in Figure 3c–e show the model fits, which reproduce the experimental trends well. The extracted fit parameters are summarized in Table 1.

**TABLE 1:** Fitted parameters from modeling the temperature‐dependent Raman modes using Equation 2 and 3, where $E_{0}$ is the mode energy at 0 K, λ is the anharmonic coupling constant, $\Gamma_{0}$ is the temperature‐independent broadening, and $\Gamma_{\mathrm{ph}}$ is the phonon‐phonon coupling strength. Reported uncertainties are the standard deviation errors from the fitting.

Parameter	E(1)	E(2)+$A_{1}$
$E_{0}$ (${\mathrm{cm}}^{-1}$)	146.18 ± 0.14	235.151 ± 0.025
λ (${\mathrm{cm}}^{-1}$)	1.6 ± 0.4	−0.75 ± 0.07
$\Gamma_{0}$ (${\mathrm{cm}}^{-1}$)	6.271 ± 0.011	
$\Gamma_{\mathrm{ph}}$ (${\mathrm{cm}}^{-1}$)	1.96 ± 0.09	

As the temperature decreases, the E(1) mode is observed to blueshift. This is a vibrational fingerprint of interchain contraction, as the restoring force associated with the bond‐shearing mode is strengthening, indicating an increased interchain coupling and a reduction in the distance between the selenium chains. In contrast, the E(2)+$A_{1}$ peak, arising from two bond‐stretching modes, redshifts upon cooling – opposite to what would be expected from simple lattice contraction. Although this redshift is smaller than the instrumental resolution, it is systematic and reproducible. This unexpected softening may result from the relaxation of grown‐in compressive strain or the development of tensile strain along the chains at lower temperatures, both of which can reduce the effective restoring forces despite thermal contraction of the Se–Se bonds. Notably, this interpretation aligns with the temperature‐dependent XRD measurements by Grosse et al. [60].

### 2.2 Photoluminescence Spectroscopy

**FIGURE 4:**
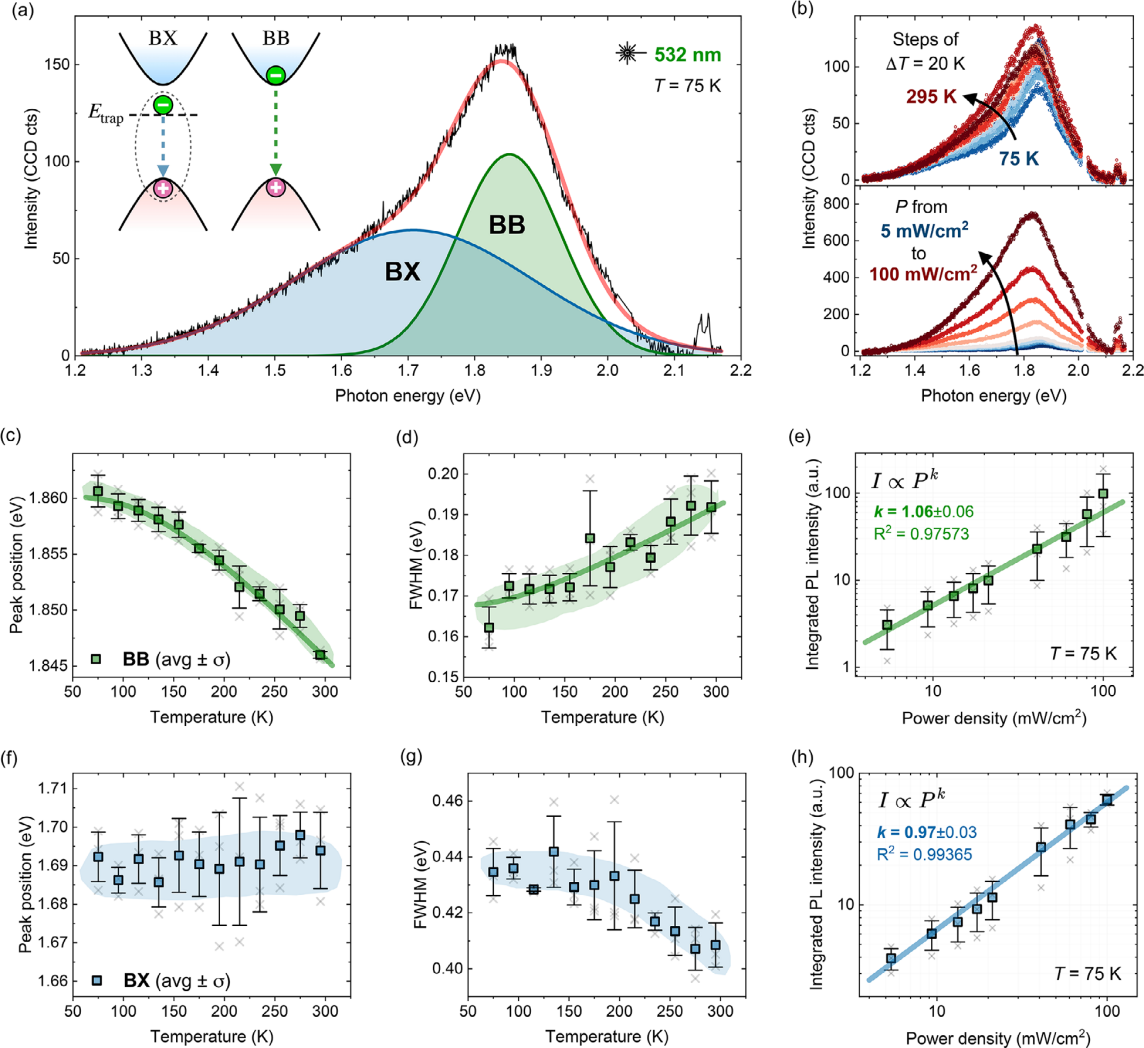
Photoluminescence characterization of the trigonal selenium thin film. (a) PL spectrum measured at 75 K, deconvoluted into band‐to‐band (BB) and defect‐bound exciton (BX) transitions, each modeled with a Gaussian peak. Inset: schematic energy band diagram illustrating the radiative recombination pathways. (b) PL spectra as a function of temperature and excitation power density. (c–e) Temperature dependence of the BB transition showing (c) peak position, (d) linewidth, and (e) integrated PL intensity as a function of excitation power at 75 K. (f–h) Corresponding data for the BX transition: (f) peak position, (g) linewidth, and (h) integrated PL intensity versus excitation power at 75 K. Solid lines in panels (c,d) and (e, h) represent fits to the Bose‐Einstein models and power‐law relations, respectively. Temperature‐dependent fits are not applied to the BX transition since Bose‐Einstein models do not adequately describe defect‐bound excitons. The results shown in panels (c–h) are averaged from three independent measurements; error bars represent one standard deviation.

We now turn to the optoelectronic characterization of the trigonal selenium thin film using photoluminescence (PL) spectroscopy. The spectrum measured at 75 K, shown in Figure 4a, is well modeled using 2 Gaussian peaks. To determine the origin of these two emission features, we apply the same deconvolution procedure to the temperature‐ and excitation power‐dependent spectra shown in Figure 4b. For the higher‐energy peak, the extracted peak position and linewidth as a function of temperature are presented in Figure 4c,d, respectively, while the integrated PL intensity as a function of excitation power is shown in Figure 4e. The corresponding results for the lower‐energy peak are shown in Figure 4f–h, respectively.

The temperature dependence of the peak position and linewidth of the higher‐energy emission feature, shown in Figure 4c,d, is well described by the Bose‐Einstein models in Equations 2 and 3, which capture the dominant electron‐phonon renormalization of the bandgap. Although lattice dilation also contributes to the overall temperature dependence of the band edge, this structural effect is implicitly included in the measured evolution and cannot be separated without independent knowledge of the thermal expansion and deformation potentials. Within this framework, the fitted parameters λ and $\hbar \bar{\omega }$ quantify the strength of the electron‐phonon interaction. In contrast, the lower‐energy peak shows minimal shift in peak position and a slight narrowing of its already broad linewidth with increasing temperature‐behaviour more indicative of defect‐related transitions and not well described by simple electron‐phonon coupling models. To further elucidate the nature of the two emission features, we next analyze their dependence on excitation power density at 75 K.

The excitation power dependence of the PL intensity is analyzed using the power‐law relation $I\propto P^{k}$, where I is the integrated PL intensity, P is the excitation power density, and k is the power‐law exponent. For both emission components, we extract k‐values close to 1. This behavior is commonly associated with band‐to‐band (BB) transitions, whereas defect‐mediated recombination typically exhibit sub‐linear behavior (k<1), and excitonic transitions result in super‐linear responses (k>1) [61, 62]. However, it is worth noting that both free‐to‐bound (FB) and defect‐bound exciton (BX) transitions can also yield k≈1, as their transition rates depend on a single type of free carrier – which scales linearly with excitation power density – and on the donor or acceptor density, which is independent of the excitation intensity. The solid lines in Figure 4c–e,h represent the model fits, and the extracted fitting parameters are summarized in Table 2.

**TABLE 2:** Fitted parameters from modeling the temperature‐ and power‐dependent PL spectra using Equations 2 and 3, where $E_{0}$ is the peak position at 0 K, λ is the electron‐phonon coupling constant, $\hbar \bar{\omega }$ is the effective phonon energy, $\Gamma_{0}$ is the temperature‐independent broadening, and $\Gamma_{\mathrm{ph}}$ is the electron‐phonon coupling strength. Reported uncertainties are the standard deviation errors from the fitting.

Parameter	BB	BX
$E_{0}$ (eV)	1.860 ± 0.001	1.688 ± 0.003
λ (meV)	43 ± 16	N/A
$\hbar \bar{\omega }$ (meV)	36 ± 8	N/A
$\Gamma_{0}$ (meV)	167 ± 5	440 ± 230
$\Gamma_{\mathrm{ph}}$ (meV)	40 ± 70	N/A
$\hbar \omega_{\mathrm{ph}}$ (meV)	30 ± 30	N/A
k	1.06 ± 0.06	0.97 ± 0.03

The power‐dependent PL measurements support the assignment of the higher‐energy emission as a band‐to‐band (BB) transition, based on its nearly linear power‐law exponent and its temperature evolution well described by Bose‐Einstein models. The lower‐energy emission exhibits a similar power‐law exponent, which alone could be consistent with either a free‐to‐bound or defect‐bound exciton transition. However, its minimal temperature‐induced shift and the slight narrowing of its already broad linewidth with increasing temperature are not characteristic of FB recombination, which typically shows stronger thermal quenching and a more pronounced redshift due to thermal ionization of the donor state. Instead, these features are more consistent with localization at extended structural defects as observed for defect‐bound excitons in monolayer ${\mathrm{MoS}}_{2}$ [63], and this interpretation aligns with early PL studies of vapor‐phase grown selenium crystals submerged in liquid hydrogen [42]. We therefore assign the two PL features to BB and BX transitions, respectively, as schematically illustrated in the inset of Figure 4a. This assignment is also consistent with recent defect calculations for trigonal selenium, which show that isolated point defects do not introduce deep electronic states, whereas extended defects such as dislocations, stacking faults, and grain boundaries could generate the mid‐gap states required to localize excitons [28]. These extended defects are therefore the most plausible origin of the broad, weakly temperature‐dependent BX emission we observe.

Finally, the characteristic phonon energy extracted from both the temperature‐dependent peak shift and linewidth broadening of the BB transition closely matches that of the E(2)+$A_{1}$ Raman peak. This suggests that one or both of these bond‐stretching vibrational modes are responsible for the dominant electron–phonon coupling driving the thermal evolution of the PL.

## 3 Discussion

### 3.1 Vibrational and Optoelectronic Response to Temperature

The thermal evolution of the vibrational and optoelectronic parameters derived from Raman and PL spectroscopy has direct implications for the optoelectronic performance potential of the selenium thin film. As the temperature is lowered, the crystal lattice is expected to contract and the electron–phonon interactions to weaken. As a direct consequence, the optical bandgap is expected to decrease, which is consistent with our observations in Figure 4c. We also observe a blueshift in the E(1) bond‐shearing mode in Figure 3d, suggesting a reduction in the distance between the helical chains and enhanced interchain coupling.

The only anomalous response is observed in the E(2)+$A_{1}$ bond‐stretching modes, which redshift slightly as the temperature decreases. Although this redshift is smaller than the instrumental resolution, its consistency across multiple measurements suggests a subtle softening of the restoring forces along the chain direction. This behavior could arise from the relaxation of grown‐in compressive strain or the buildup of tensile stress along the chains at lower temperatures, and may contribute to the PL linewidth broadening by modifying exciton confinement potentials and/or facilitating the formation of extended structural defects. Since the crystal grains are randomly oriented, this strain cannot be attributed solely to lattice mismatch with the substrate. Instead, it likely arises from internal stress accumulated during thermal annealing and cooling during crystallization, which may promote the formation of dislocations, stacking faults, or additional grain boundaries. Direct quantification of such strain would require temperature‐dependent diffraction measurements, which fall outside the scope of the present study but represent an important direction for future work. This interpretation is consistent with the deep‐level transient spectroscopy (DLTS) study by Chen et al. [2], which attributed the dominant trap states in selenium and selenium‐tellurium alloy thin films to extended, rather than point‐like, defects. It also aligns with the combined computational and experimental study by Kavanagh et al. [28], which showed that intrinsic point defects in trigonal selenium do not form killer recombination centers.

Overall, these results demonstrate that the vibrational response and carrier dynamics of trigonal selenium are governed not by intrinsic point defects but by strain fields and extended structural imperfections, emphasizing the importance of microstructural control in achieving high optoelectronic quality.

### 3.2 Cross‐Laboratory Comparison and Sensitivity to Processing Conditions

To evaluate how sensitive the structural quality of selenium thin films is to subtle, unintentional variations in processing, we compare samples fabricated via the standardized two‐step synthesis route – thermal evaporation of amorphous selenium followed by thermal annealing in air – carried out in different laboratories. Specifically, we examine the thermal evolution of the PL spectra of our sample from UPC alongside a sample fabricated at DTU under nominally identical conditions, i.e., using identical source materials as well as the same nominal evaporation and annealing temperatures in both laboratories. As shown in Figure S2, the DTU film features larger crystal grains, while both samples exhibit similar PL spectral shapes, including a defect‐bound exciton and a band‐to‐band transition. Although the DTU group previously reported no detectable PL at room temperature, our measurements confirm its presence, emphasizing the effectiveness of closed‐space encapsulation in preventing surface degradation and the advantage of using a microscope objective to collect PL over a larger solid angle.

A quantitative comparison of the BB peak parameters reveals that the peak position is ≈5 meV lower at low temperatures in the DTU sample, and the electron‐phonon coupling strength is ≈20−21 meV, which is about half the value extracted from the UPC sample. These differences may be explained by the ultra‐thin tellurium nucleation layer, which has been observed to interdiffuse into the selenium layer during the crystallization [64]. Given that selenium‐tellurium alloys exhibit a reduced optical bandgap with increasing Te content [24, 25], the slightly lower bandgap in the DTU film may indicate greater Te incorporation, which could in turn influence lattice dynamics and weaken coupling to bond‐stretching modes. Alternatively, seemingly minor differences in thermal processing – such as marginally higher peak temperatures during the rapid thermal annealing step or variations in the cooling rate – could also contribute to the reduced electron–phonon coupling, particularly given the observed increase in grain size. Such sensitivity to annealing and cooling conditions strongly suggests that microstructural relaxation pathways differ between laboratories, leading to measurable changes in coupling to high‐frequency vibrational modes. Regardless of the precise origin, these results demonstrate that the coupling strength of the electrons to the high‐frequency vibrational modes is not intrinsic to the material, but depend strongly on the microstructure or the thin film and the processing history, emphasizing the need for precise control over synthesis conditions to optimize optoelectronic performance.

Notably, the DTU samples have demonstrated the highest reported open‐circuit voltage of 0.99 V in photovoltaic devices [27] compared to 0.86 V for the UPC samples, highlighting the device implications of improving the optoelectronic quality of the absorber. The reduced electron‐phonon coupling strength and larger grain size observed in the DTU films point to lower densities of structural defects and fewer pathways for non‐radiative recombination, supporting a larger quasi‐Fermi level splitting under illumination. This reduction in non‐radiative recombination channels is consistent with longer effective carrier lifetimes and higher radiative efficiency [38, 48]. In contrast, the stronger coupling to high‐frequency bond‐stretching modes in the UPC films, combined with their smaller grain size, suggests enhanced carrier scattering and a greater prevalence of extended defects. These factors facilitate non‐radiative recombination and limit the quasi‐Fermi level splitting, thereby suppressing the open‐circuit voltage. Overall, the microstructural differences reflected in the optical parameters provide a mechanistic explanation for the open‐circuit voltage disparity between the two laboratories' devices.

### 3.3 Defects and Recombination Pathways

The interplay between vibrational and optoelectronic properties has a strong influence on overall device performance. Our results show that structural disorder in selenium thin films is not intrinsic but highly sensitive to subtle variations in processing. These extended defects likely act as killer non‐radiative recombination centers. Because such defects are not necessarily radiative, their impact may not be fully captured by PL measurements alone. However, we do observe a defect‐bound exciton emission feature. Prior work by Nielsen et al. [65] identified an acceptor‐like trap with a similar energy level, possibly corresponding to the (+1/0) charge transition level of selenium vacancies [28]. While this lower‐energy exciton‐related emission is unlikely to represent the primary non‐radiative loss mechanism responsible for the large open‐circuit voltage deficit, it may still contribute to the broad absorption onset commonly observed in both EQE and absorption spectra. This would help explain the persistent discrepancy between our PL‐inferred optical bandgaps (1.84–1.86 eV), and the photovoltaic bandgaps (∼1.95 eV) derived from the inflection point of the EQE [34, 50, 66, 67]. This discrepancy is likely a result of sub‐bandgap absorption from band tailing induced by traps and/or structural disorder. Earlier work by Chen et al. [68] also reported strong trapping of free excitons in selenium and proposed that such trapping could account for significant non‐radiative recombination losses at higher temperatures.

Together, these observations highlight that the dominant recombination pathways in trigonal selenium thin films are controlled by extended defects and local structural distortions, reinforcing the need for defect‐aware growth strategies and microstructural engineering to close the voltage deficit in selenium photovoltaics.

### 3.4 Implications for Device Optimization

The combination of phonon band structure effects, strain‐driven vibrational softening, extended‐defect formation, and processing‐dependent electron–phonon coupling reveals a coherent picture in which optoelectronic quality is governed by microstructure rather than intrinsic limitations of trigonal selenium. This offers a clear route toward performance improvements: minimizing extended defects, controlling strain, and tailoring phonon dispersion through alloying, nanostructuring, or optimized annealing protocols.

## 4 Conclusion

In summary, we have used temperature‐dependent Raman and PL spectroscopy to investigate grown‐in stress, vibrational dynamics, and electron–phonon interactions in selenium thin films synthesized in different laboratories. Our results underscore the critical relation between the microstructural and optoelectronic quality, which ultimately governs device performance. We hypothesize that the structural disorder may facilitate the formation of extended defects, which are believed to be the dominant non‐radiative recombination pathways limiting carrier lifetime and open‐circuit voltage in selenium solar cells. A key finding is that this structural disorder is not intrinsic to the material, but is highly sensitive to subtle processing variations. Notably, films prepared using nominally similar methodologies in different labs exhibit marked differences in short‐range structural disorder, which significantly influences the electron‐phonon coupling strength and overall optoelectronic quality. This comparative analysis confirms that short‐range electron–phonon interactions are tunable through precise control of synthesis parameters, reflecting variations in structural order and crystalline quality.

For future work, we suggest that processing strategies emphasizing careful thermal management during crystallization could help minimize anisotropic strain and suppress the formation of extended defects. Such stress may arise not only from growth conditions and substrate interactions, but also from differential thermal contraction during the cooling phase, particularly in thin films where temperature gradients and substrate anchoring can induce significant levels of grown‐in stress. Post‐deposition treatments, such as additional annealing protocols designed to relax residual stress and reduce structural disorder without introducing new defects, represent another promising route for improving material quality. Additionally, we demonstrate that temperature‐dependent Raman and PL spectroscopy is a fast, non‐destructive, and effective toolkit for assessing structural and optoelectronic quality even in randomly oriented polycrystalline films. These techniques enable the detection of strain signatures, shifts in bond‐stretching and ‐shearing modes, and insight into the nature of radiative recombination channels. When integrated with other device‐level techniques – such as temperature‐dependent current‐voltage measurements, transient photoconductivity, and capacitance‐based defect spectroscopy – this multimodal approach provides a powerful platform to explore how the microscopic structure and defect landscapes influence macroscopic device performance.

## 5 Methods

### 5.1 Materials

Fluorine‐doped tin oxide (FTO)‐coated glass substrates (TEC 15, ∼13 Ω/sq) and tellurium pellets (99.999%, metals basis) were purchased from Sigma‐Aldrich. Selenium powder (200 mesh, 99.999%, metals basis) was obtained from Thermo‐Scientific. Gold (99.99%) for back contacts was purchased from Neyco.

### 5.2 Fabrication of Selenium Samples

FTO‐coated glass substrates (25 mm × 25 mm × 2.2 mm) were ultrasonically cleaned sequentially in acetone, isopropanol, and Milli‐Q water, then dried using a nitrogen gun. The absorber layer was deposited using a Kenosistec co‐evaporation system at a base pressure below $5\times 10^{-7}$ mbar. First, an ultrathin (∼1 nm) tellurium layer was evaporated, immediately followed by the deposition of 300 nm of selenium at an evaporation rate of 10–14 nm/min, with no substrate heating. The as‐deposited amorphous selenium absorber was then crystallized by annealing the samples in air on a hotplate at 200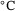 (measured by the hotplate's internal thermocouple) for 5 minutes, before being returned to vacuum. Gold back contacts were deposited in the same system at a rate of 1–2 Å/s through a shadow mask, resulting in an active cell area of 7 ${\mathrm{mm}}^{2}$. The fabrication process for the samples from DTU, presented in the Supporting Information, has been described in detail elsewhere [27].

### 5.3 Raman and Photoluminescence

Raman and photoluminescence (PL) spectra were acquired using a WITec alpha300 R confocal Raman microscope in a backscattering configuration. The measurements were performed in an optical cryostat maintained at a base pressure of 10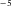 mbar, with the sample chuck cooled using liquid nitrogen. The temperature of the sample chuck was monitored using a thermocouple. After the desired setpoint temperature had been reached, the system was left to stabilize for an additional 30 min to ensure thermal equilibrium between the sample and the stage. A 532 nm excitation laser was focused onto the sample using a 50x long working distance microscope objective (NA = 0.55), resulting in a spot diameter of approximately 1.2 μm. The backscattered light was collected and analyzed using a thermoelectrically cooled CCD spectrometer equipped with a holographic grating (150 g/mm for PL and 1800 g/mm for Raman). For both PL and Raman measurements, spectra were averaged over 100 μm line scans comprising 30 points, with an integration time of 10 s per point, to improve the signal‐to‐noise ratio and reduce the influence of local sample inhomogeneities. Raman peak positions were calibrated using the reference silicon peak at 520 ${\mathrm{cm}}^{-1}$. All raw spectra and acquisition parameters are provided in the Supporting Information.

### 5.4 Additional Characterization

Scanning electron microscopy (SEM) images were acquired using a Zeiss Auriga field‐emission microscope operated at an acceleration voltage of 5 kV, with working distances between 3 and 5 mm. X‐ray diffraction (XRD) patterns were collected using a Bruker D8 Advance diffractometer equipped with a copper X‐ray tube (40 kV, 40 mA) and a Sol‐X detector. Measurements were conducted in Bragg‐Brentano geometry, and the detector was configured with a discriminator to suppress the Cu $K_{\beta }$ line and minimize fluorescence background.

### 5.5 Computational Details

Calculations were performed using Density Functional Theory (DFT) through the Vienna Ab Initio Simulation Package (VASP) [69, 70] with the projector‐augmented wave (PAW) pseudopotentials [71]. The range‐separated screened hybrid DFT functional of Heyd, Scuseria and Ernzerhof (HSE06) [72] was used for atomic relaxation and displacement calculations, along with the D3 correction [73] with the zero‐damping function, to account for van der Waals (vdW) type dispersion forces – found to significantly impact the structural properties of solid‐state selenium [28]. A plane wave energy cutoff of 300eV and k‐point density of 0.42 Å^−1^ (4×4×4 for the 3‐atom $P3_{1}21$ unit cell) were used, found to give total energies converged to within 1meV/atom using vaspup2.0 [74]. phonopy [75] and ThermoParser [76] were used to setup and parse the phonon calculations, which used a 4×4×4 supercell of the primitive trigonal selenium unit cell. The ionic dielectric response and Born effective charges were calculated using linear response theory under finite electric fields, also with the HSE06+D3 functional, in order to compute the non‐analytical correction (NAC) to the phonon dispersion. See Ref. [28] for further computational details.

## Author Contributions

R.S.N. was responsible for data acquisition and formal analysis, wrote the original draft, contributed to funding acquisition, and co‐managed the project. A.G.M. contributed equally to data acquisition. A.T. and O.S.B. synthesized the main samples and performed XRD and SEM measurements. S.R.K. developed the computational methodology. D.O.S. and A.W. conceptualized the computational aspects and contributed to funding acquisition. E.S. and M.P. supervised A.T. and O.S.B. and contributed to funding acquisition. M.D. did conceptualization, supervision, provided resources, and co‐managed the project. All authors contributed to discussions and to writing – review and editing.

## Conflicts of Interest

The authors declare no conflict of interest.

## Data availability Statement

The data that support the findings of this study are openly available in Zenodo at https://doi.org/10.5281/zenodo.16639518, reference number 16639518.

## Supporting information


**Supporting File 1**: smtd70463‐sup‐0001‐SuppMat.pdf.

**Supporting File 2**: smtd70463‐sup‐0002‐FigureS1‐S6.zip.
