# Supplementary material for: Spectroscopic Signatures of Structural Disorder and Electron‐Phonon Interactions in Trigonal Selenium Thin Films for Solar Energy Harvesting
Source: Small Methods. 2026 Jan 14;10(3):e01841. doi: 10.1002/smtd.202501841 (PMC12893303; doi:10.1002/smtd.202501841)
Supplement: Supplementary file 1 — Supporting File 1: smtd70463‐sup‐0001‐SuppMat.pdf. [file SMTD-10-e01841-s001.pdf]

# SUPPORTING INFORMATION

## Spectroscopic Signatures of Structural Disorder and Electron-Phonon Interactions in Trigonal Selenium Thin Films for Solar Energy Harvesting

Rasmus S. Nielsen,[1, *] Axel G. Medaille,[2, 3] Arnau Torrens,[2, 3] Oriol Segura-Blanch,[2, 3] Seán R. Kavanagh,[4] David O. Scanlon,[5] Aron Walsh,[6, 7] Edgardo Saucedo,[2, 3] Marcel Placidi,[2, 3] and Mirjana Dimitrievska[1, †]

[1] *Nanomaterials Spectroscopy and Imaging, Transport at Nanoscale Interfaces Laboratory, Swiss Federal Laboratories for Material Science and Technology (EMPA), Ueberlandstrasse 129, 8600 Duebendorf, Switzerland*

[2] *Universitat Politècnica de Catalunya (UPC), Photovoltaic Lab - Micro and Nano Technologies Group (MNT), Electronic Engineering Department, EEBE, Av Eduard Maristany 10-14, Barcelona 08019, Spain*

[3] *Universitat Politècnica de Catalunya (UPC), Barcelona Centre for Multiscale Science & Engineering, Av Eduard Maristany 10-14, Barcelona 08019, Spain*

[4] *Harvard University Center for the Environment, Cambridge, Massachusetts 02138, United States*

[5] *School of Chemistry, University of Birmingham, Birmingham B15 2TT, UK*

[6] *Thomas Young Centre and Department of Materials, Imperial College London, London SW7 2AZ, UK*

[7] *Department of Physics, Ewha Womans University, Seoul 03760, Korea*

---

* Electronic mail: raniel@dtu.dk

† Electronic mail: mirjana.dimitrievska@empa.ch

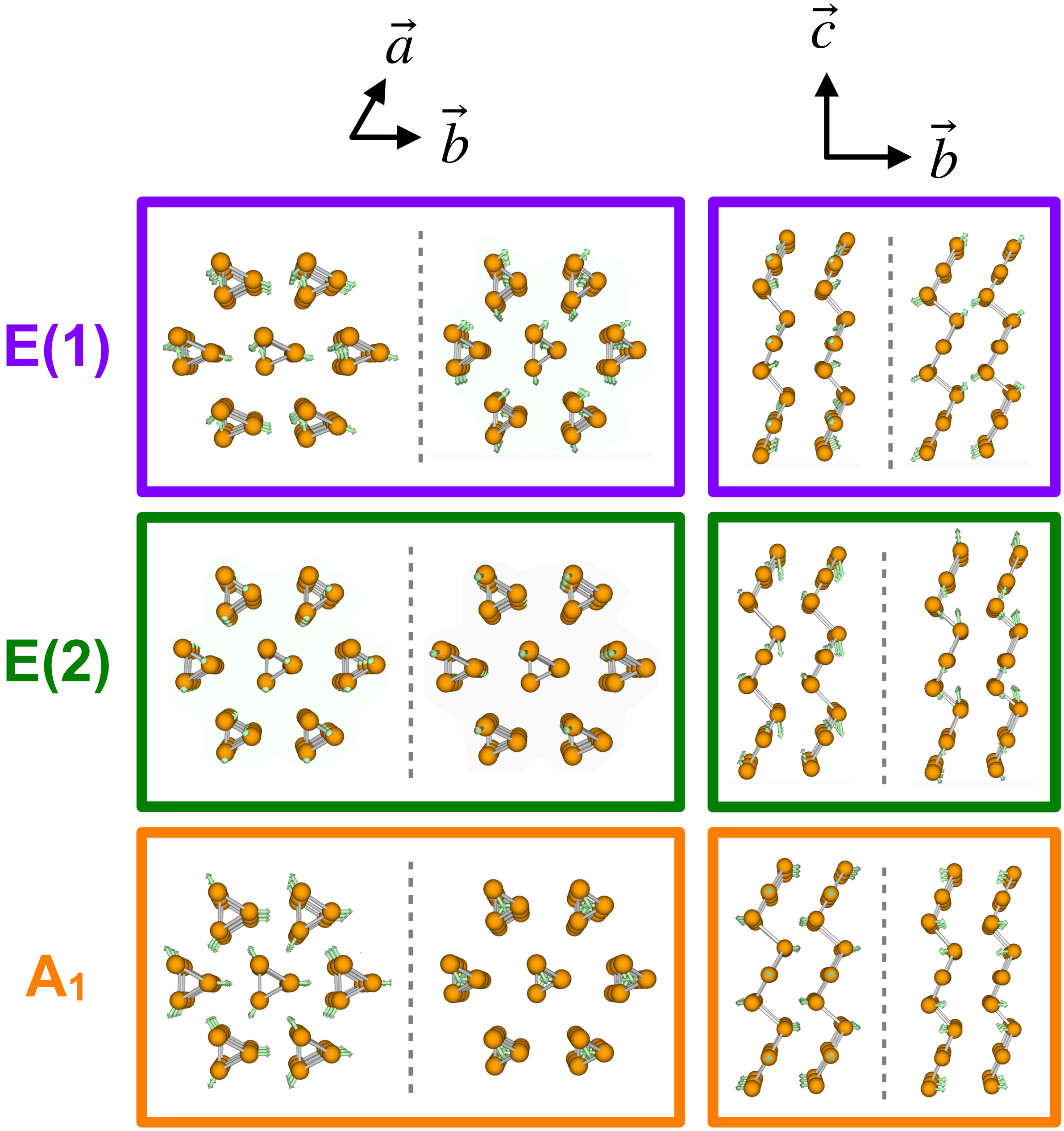


FIG. S1. Phonon displacements viewed along different lattice directions.

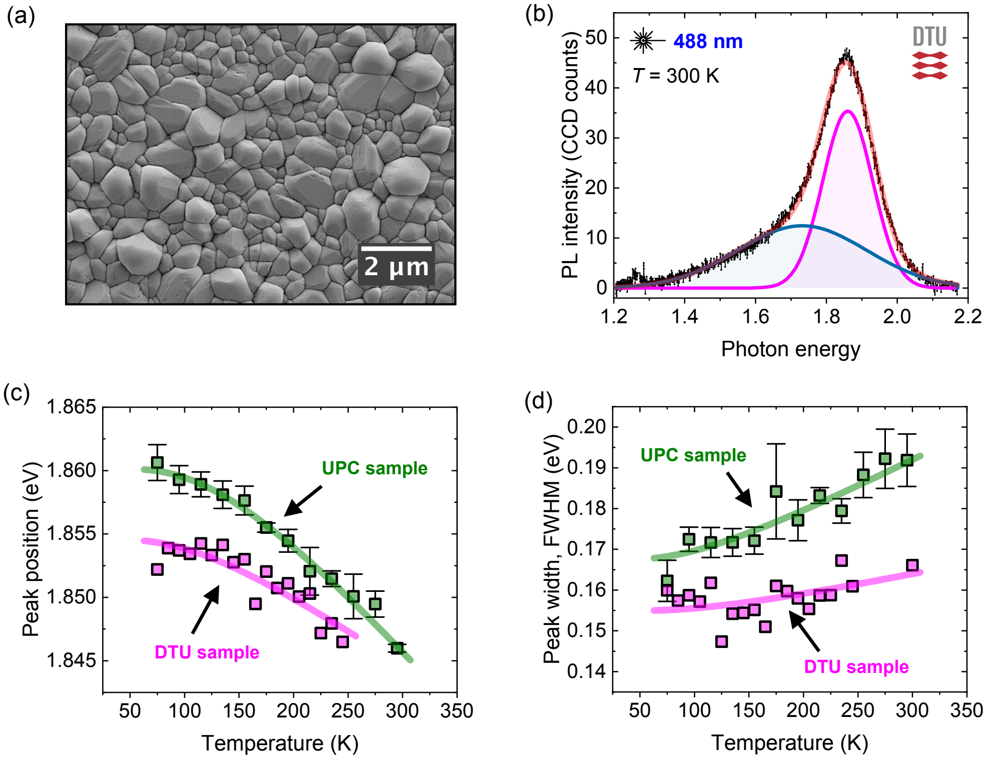


FIG. S2. Comparison of selenium thin films synthesized at DTU and UPC. (a) Top-view SEM image of the DTU sample showing larger crystal grains compared to the UPC sample. (b) Room-temperature PL spectrum of the DTU sample, revealing the same spectral features as the UPC sample – namely, the band-to-band (BB) and defect-bound exciton (BX) transitions. However, the BB emission in the DTU sample is sharper and more dominant than the BX emission. (c) Temperature-dependence of the BB peak position for both samples. The DTU sample exhibits a slightly lower optical bandgap at low temperatures ($E_0 = 1.855$ eV), but the reduced strength of electron-phonon interactions causes both samples to converge to approximately the same bandgap at room temperature. (d) Temperature-dependence of the full width at half maximum (FWHM) of the BB peak. The fitted electron-phonon coupling strengths – $\lambda = 21.6$ meV from the peak shift and $\Gamma_{\mathrm{ph}} = 19.6$ meV from the peak broadening – are approximately half the values fitted for the UPC sample. The fitted inhomogeneous broadening of the DTU sample is $\Gamma_0 = 0.155$ eV.

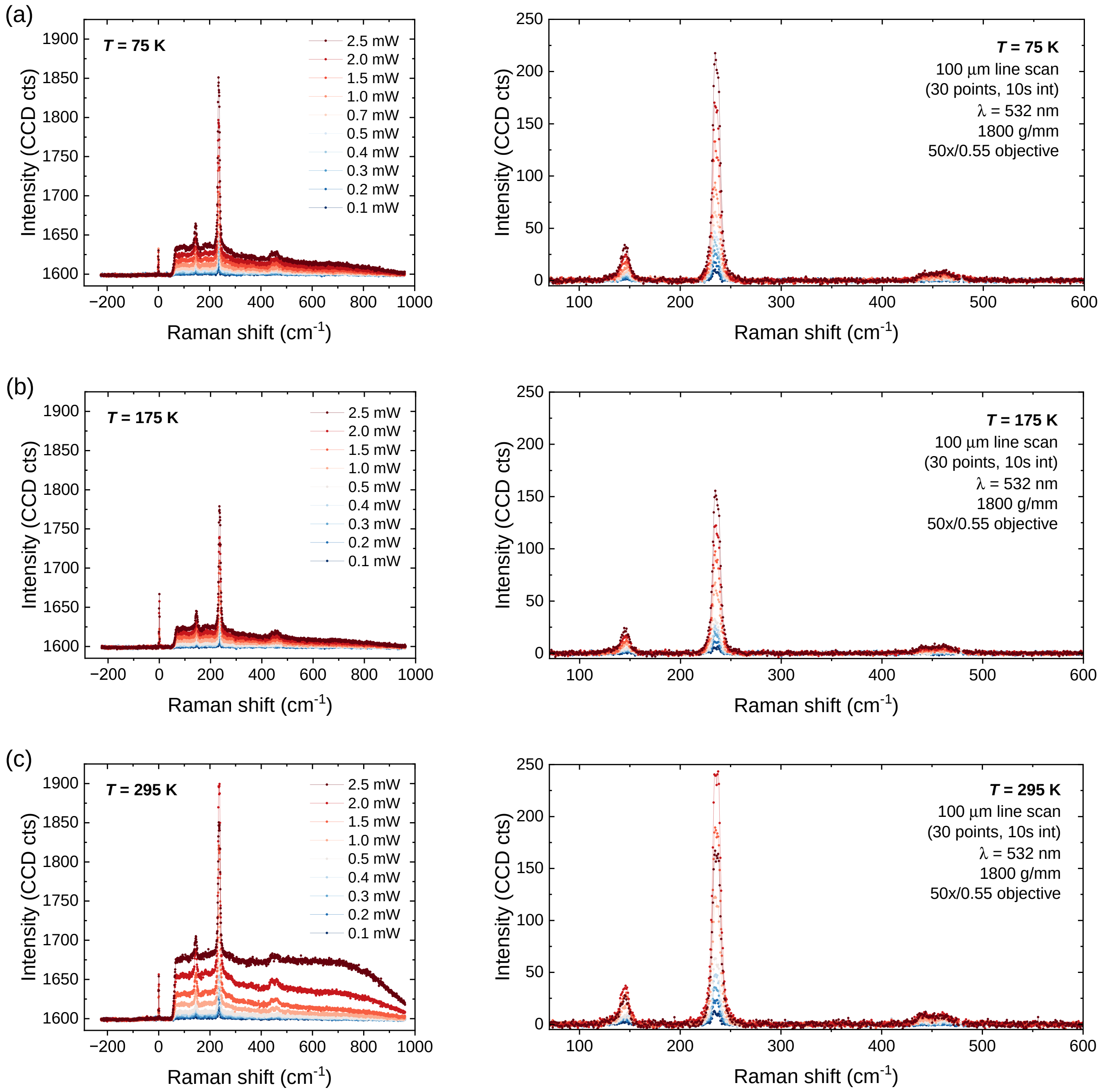


FIG. S3. Raman spectra of selenium thin films measured under varying excitation powers at (a) 75 K, (b) 175 K, and (c) 295 K. Raw spectra (left) and background-subtracted spectra (right) are shown for each temperature. Acquisition parameters are indicated in the plots.

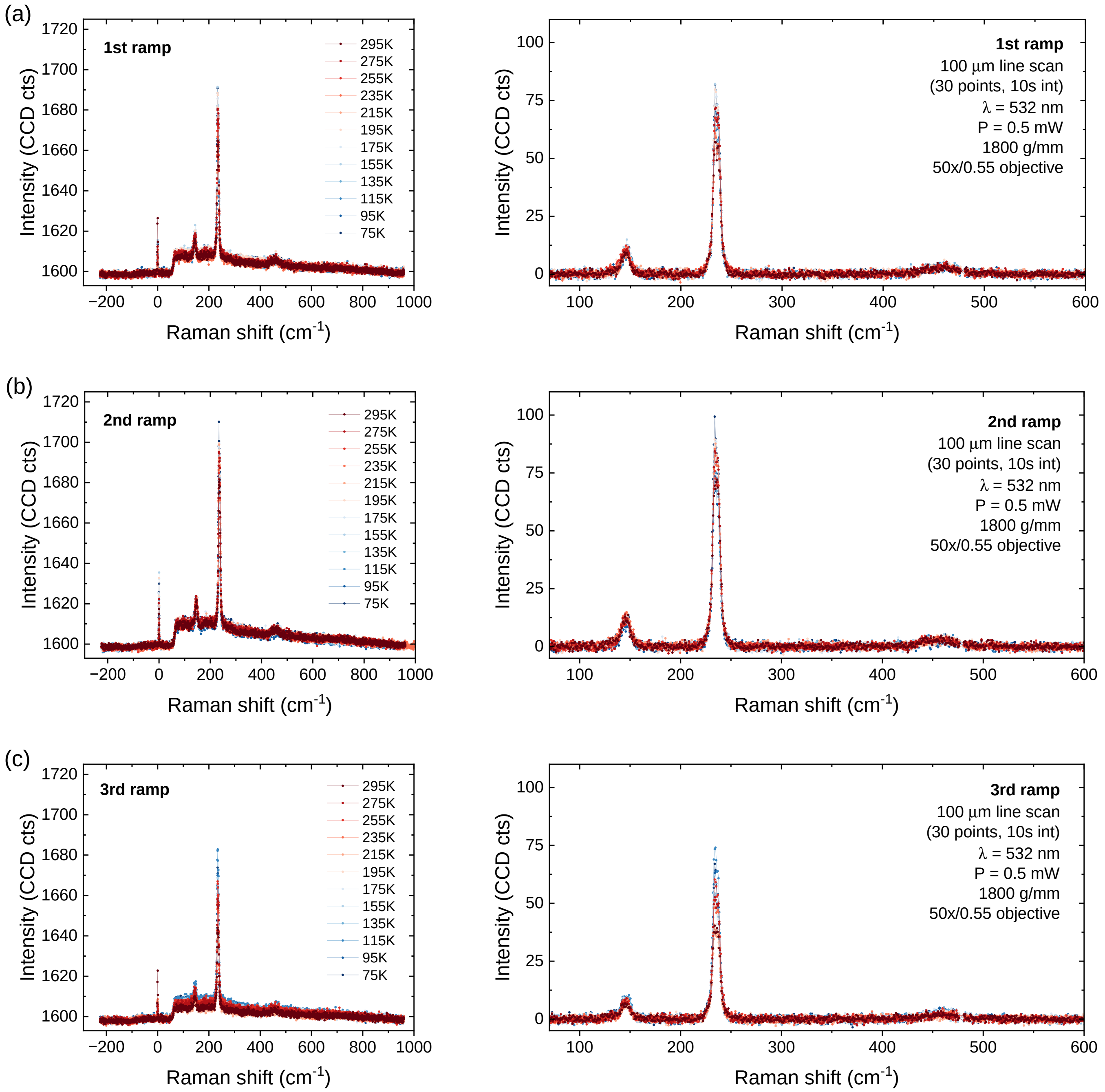


FIG. S4. Raman spectra of selenium thin films measured as a function of temperature: (a) first ramp, (b) second ramp, and (c) third ramp, each acquired at a different area on the sample. Raw spectra (left) and background-subtracted spectra (right) are shown for each measurement. Acquisition parameters are indicated in the plots.

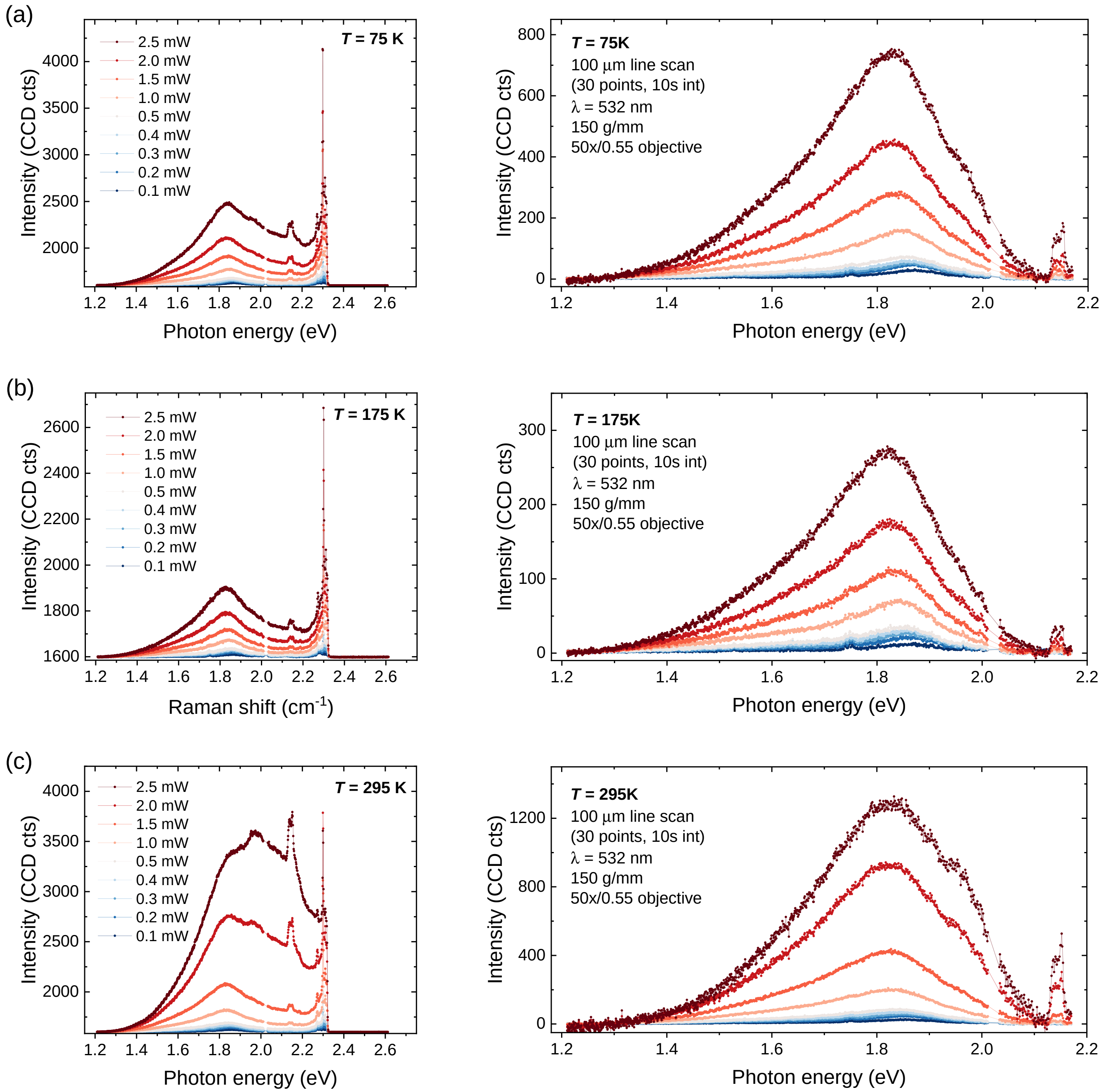


FIG. S5. Photoluminescence spectra of selenium thin films measured under varying excitation powers at (a) 75 K, (b) 175 K, and (c) 295 K. Raw spectra (left) and background-subtracted spectra (right) are shown for each temperature. Acquisition parameters are indicated in the plots.

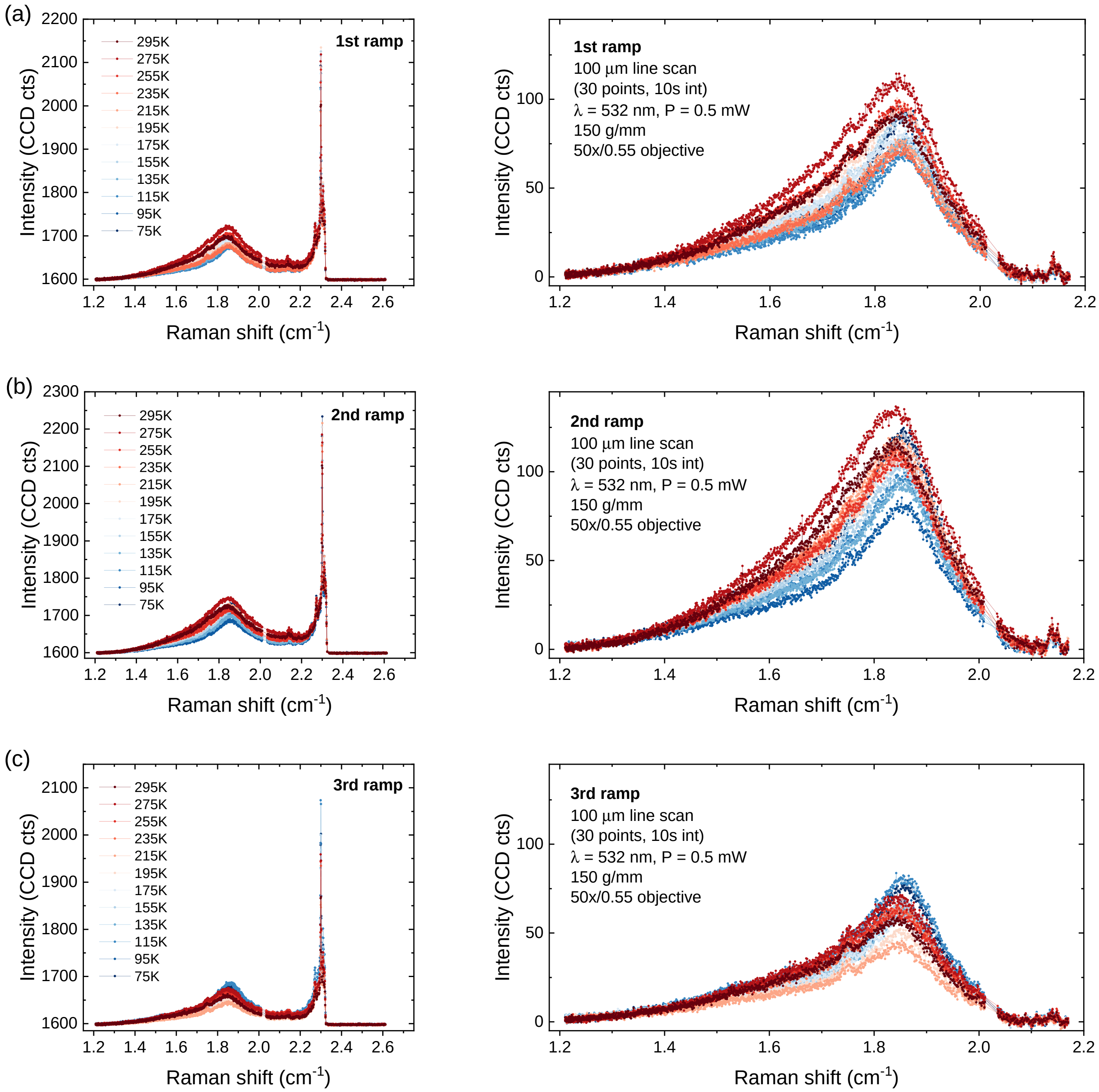


FIG. S6. Photoluminescence spectra of selenium thin films measured as a function of temperature: (a) first ramp, (b) second ramp, and (c) third ramp, each acquired at a different area on the sample. Raw spectra (left) and background-subtracted spectra (right) are shown for each measurement. Acquisition parameters are indicated in the plots.
