# Supplementary figures and images for: Spectroscopic Signatures of Structural Disorder and Electron‐Phonon Interactions in Trigonal Selenium Thin Films for Solar Energy Harvesting
Source: Small Methods. 2026 Jan 14;10(3):e01841. doi: 10.1002/smtd.202501841 (PMC12893303; doi:10.1002/smtd.202501841)

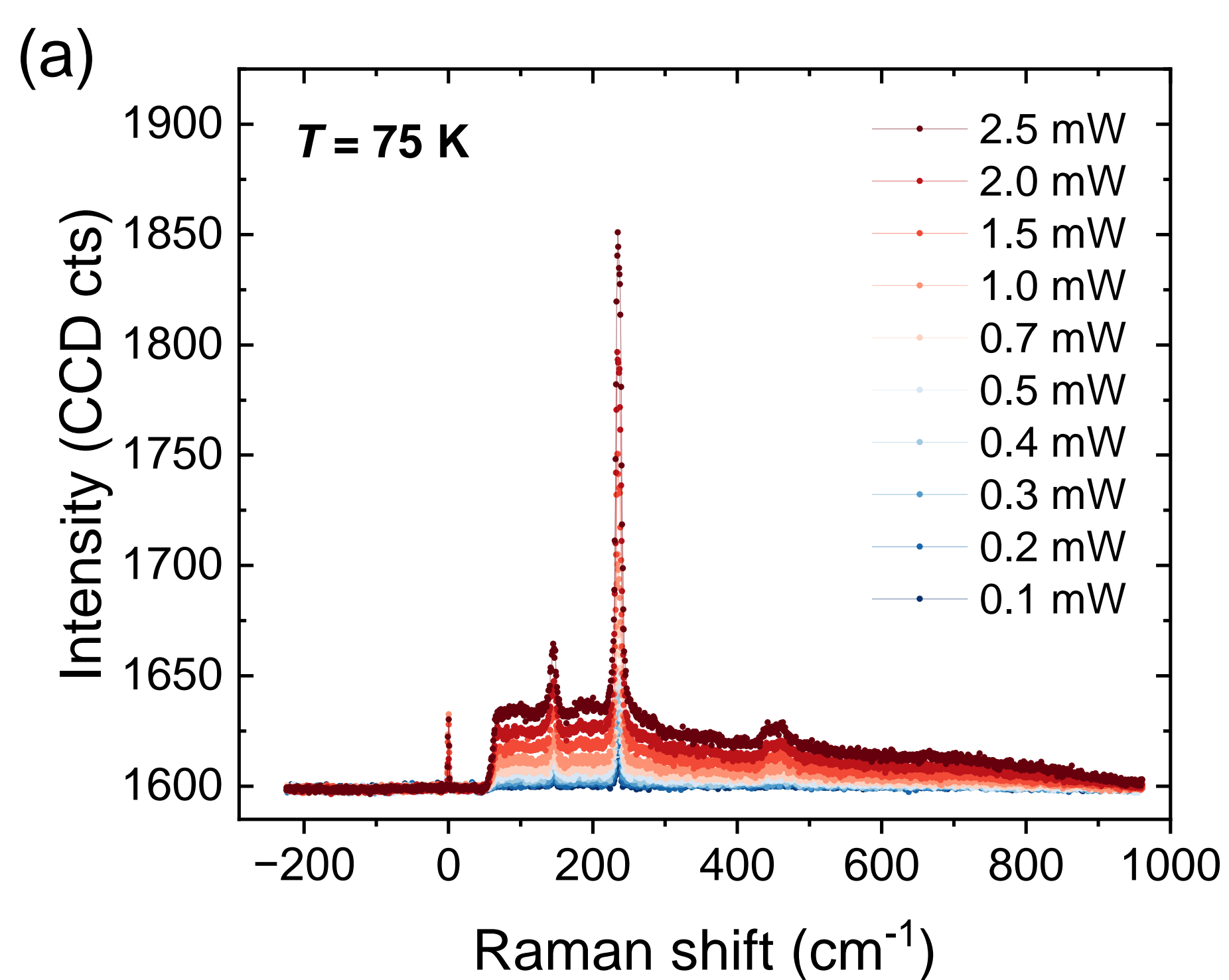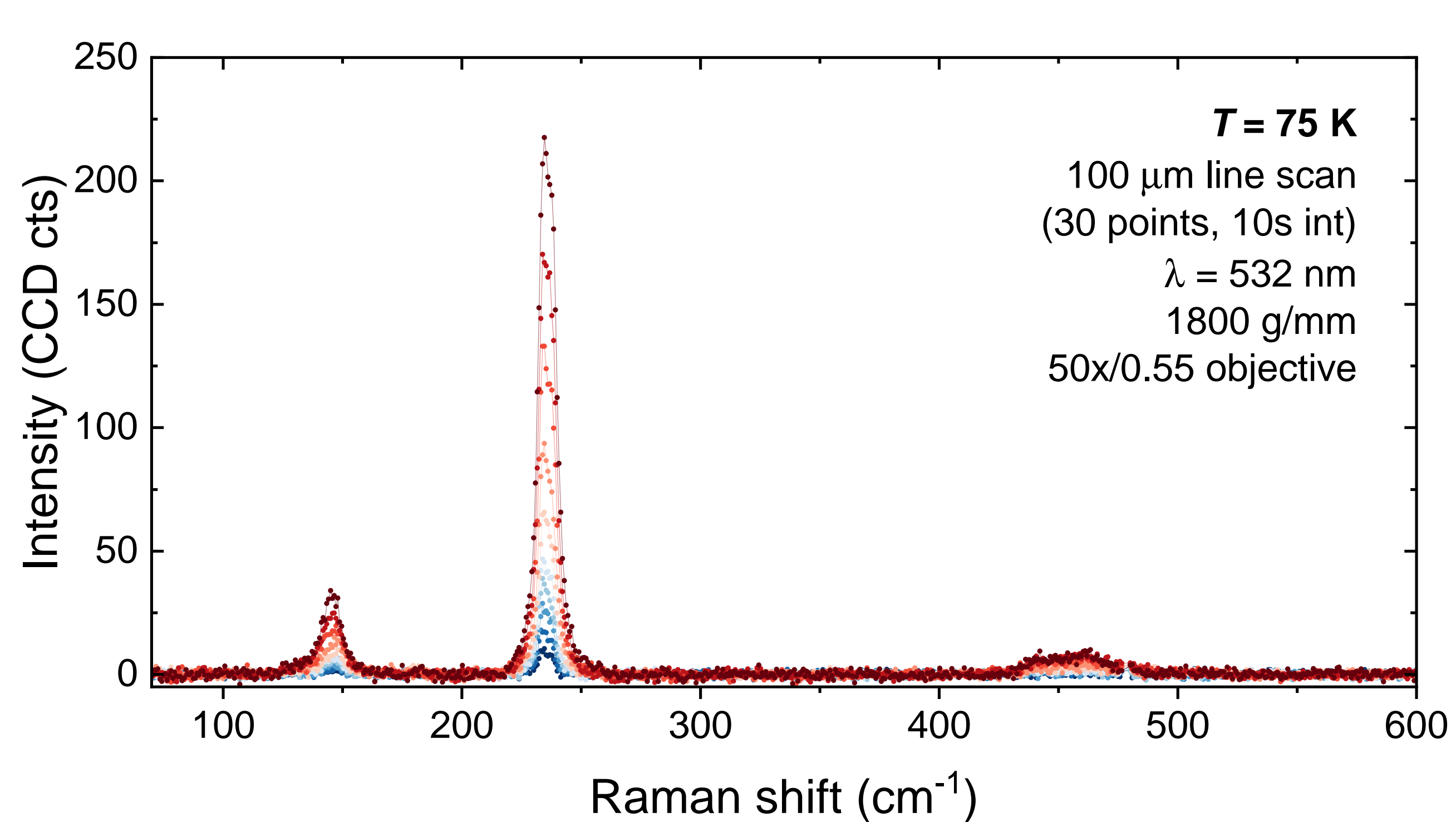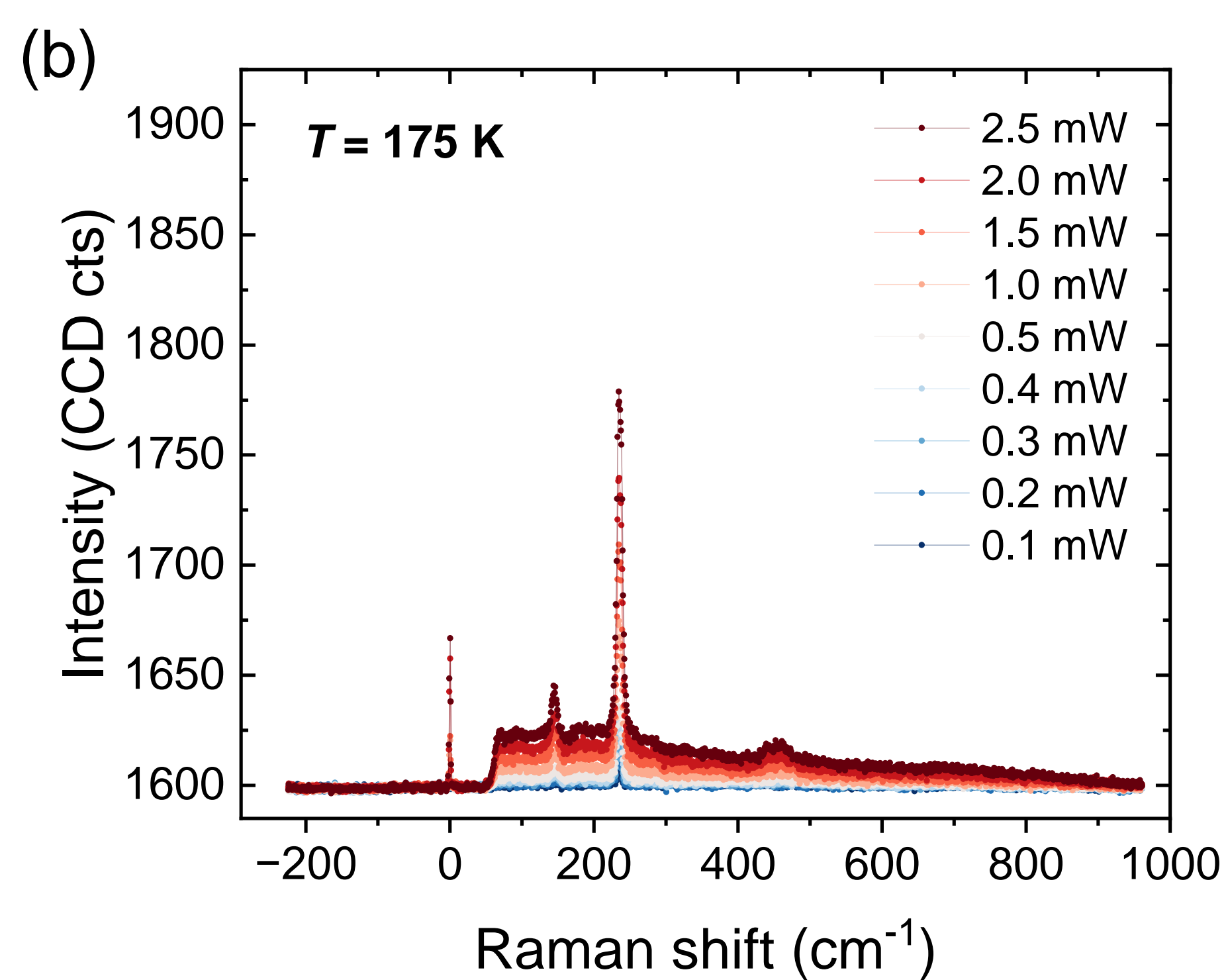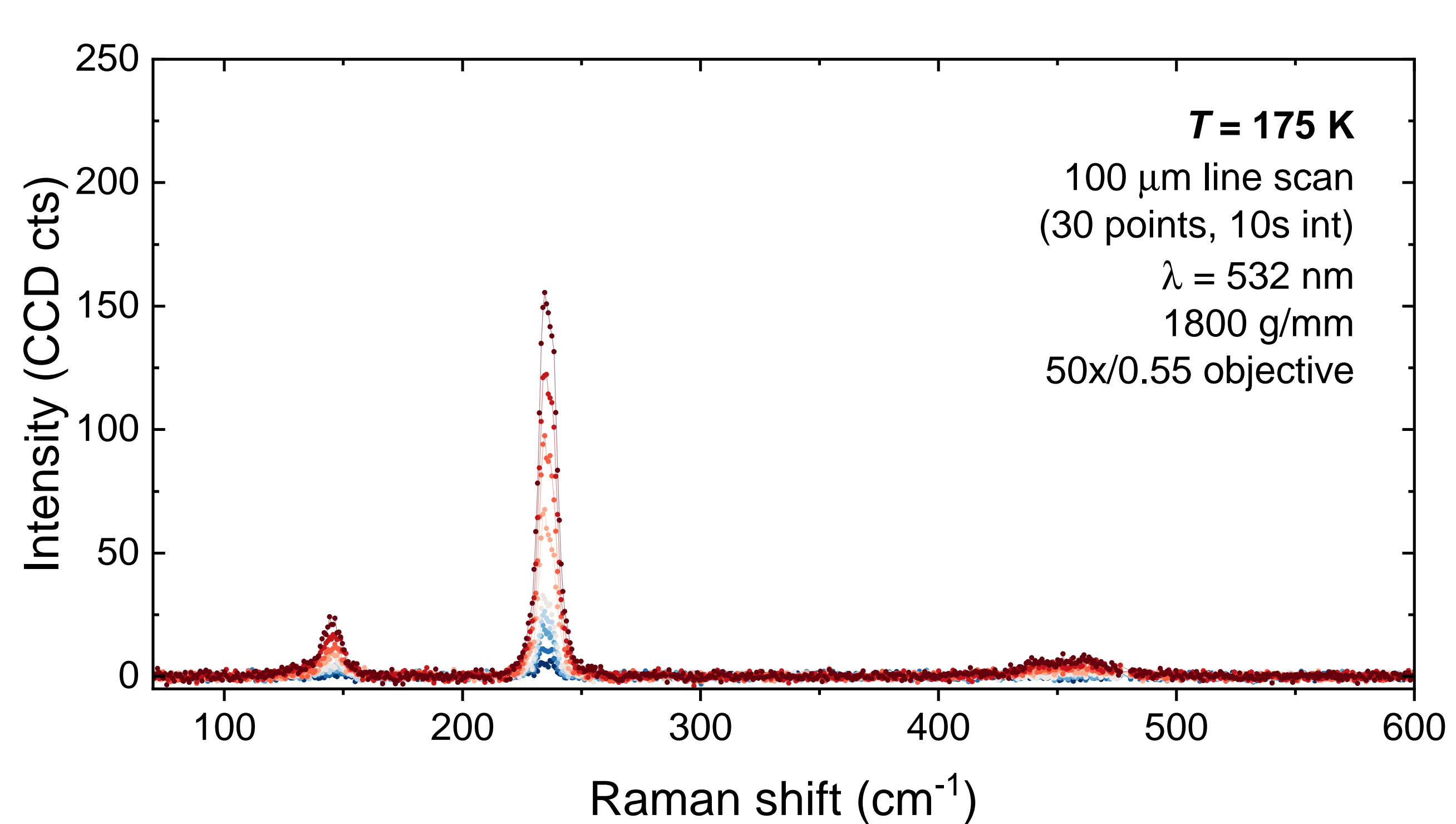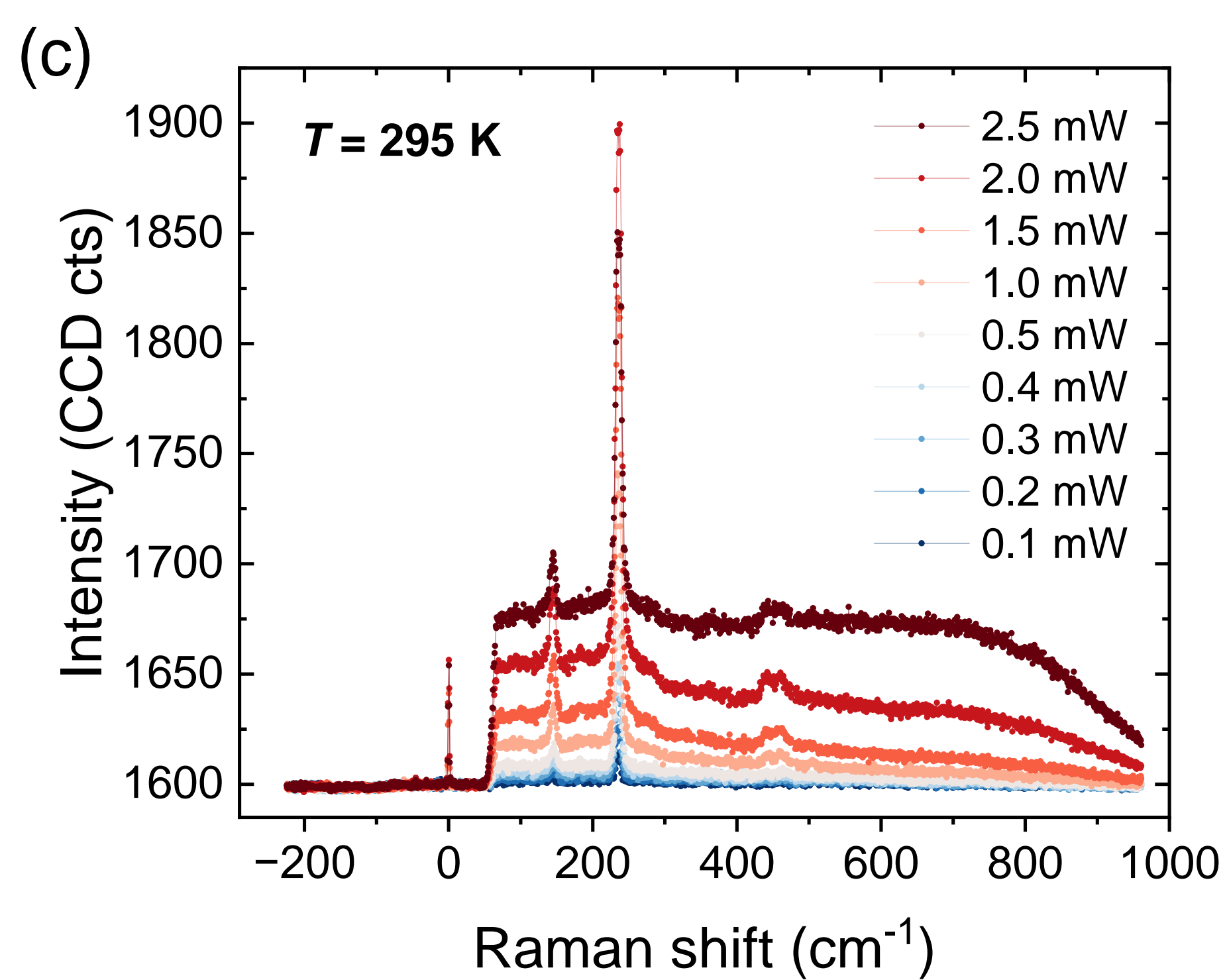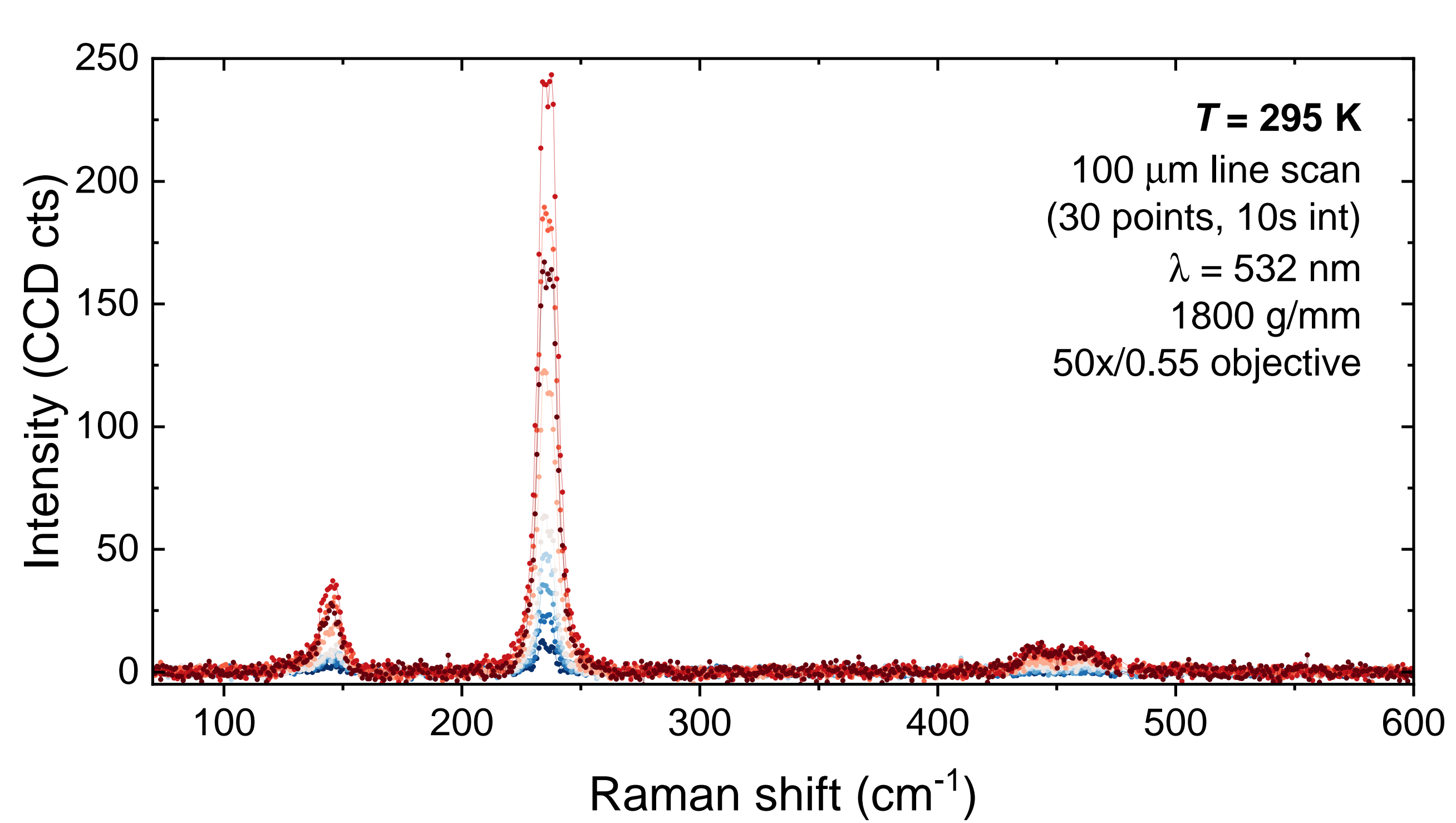

Supplement: Supplementary file 2 — Supporting File 2: smtd70463‐sup‐0002‐FigureS1‐S6.zip. [file SMTD-10-e01841-s002.zip › FigureS1.pdf]

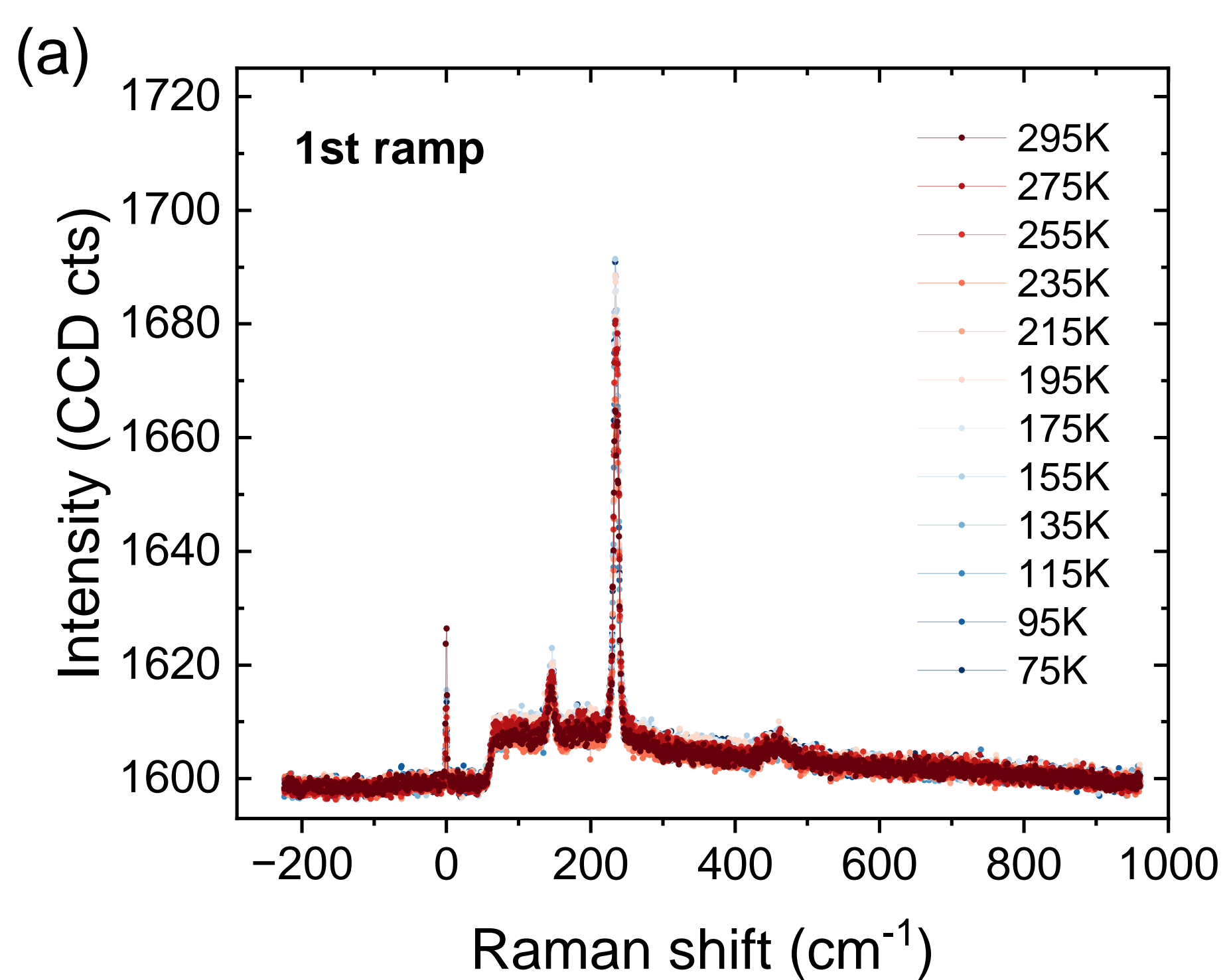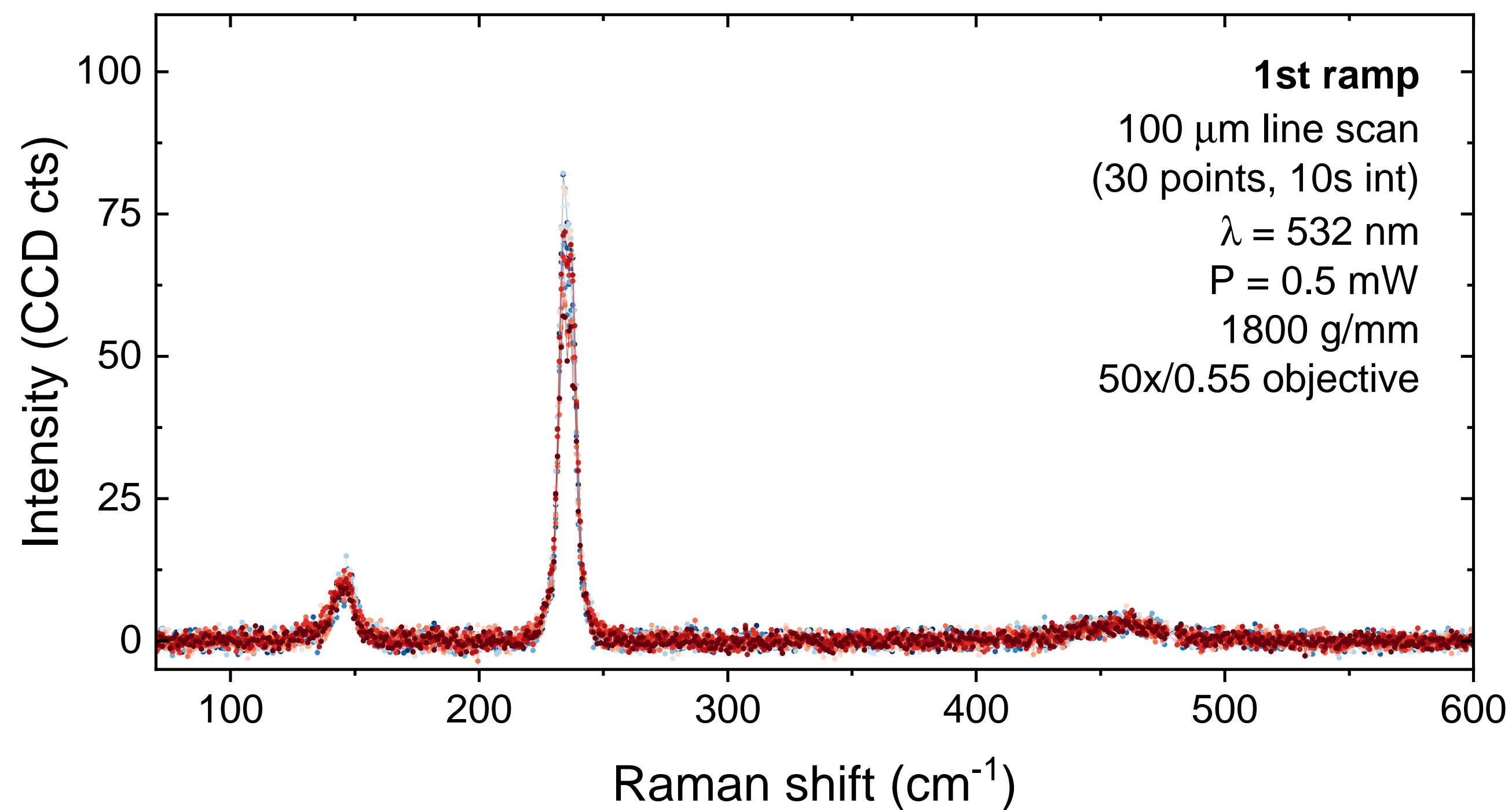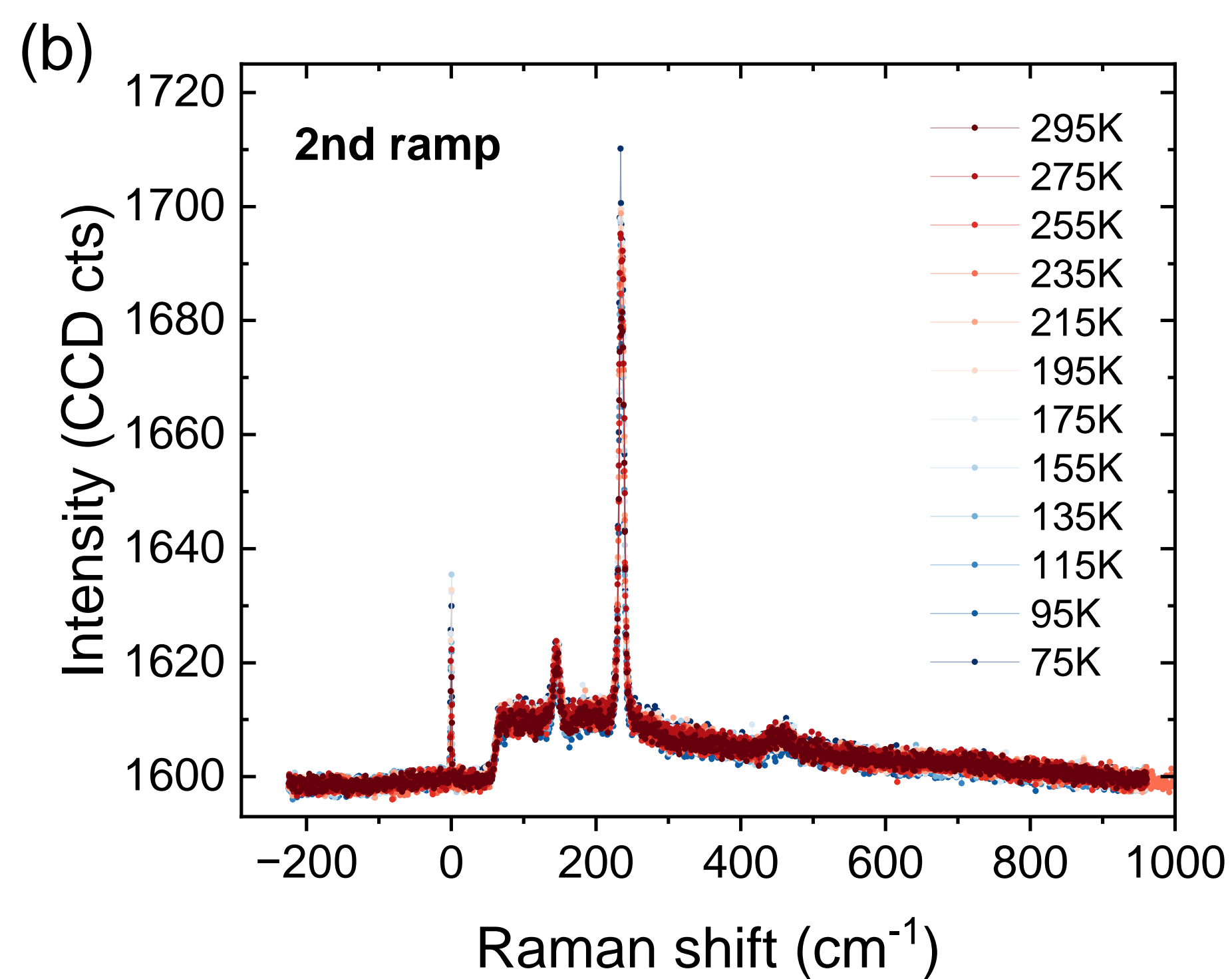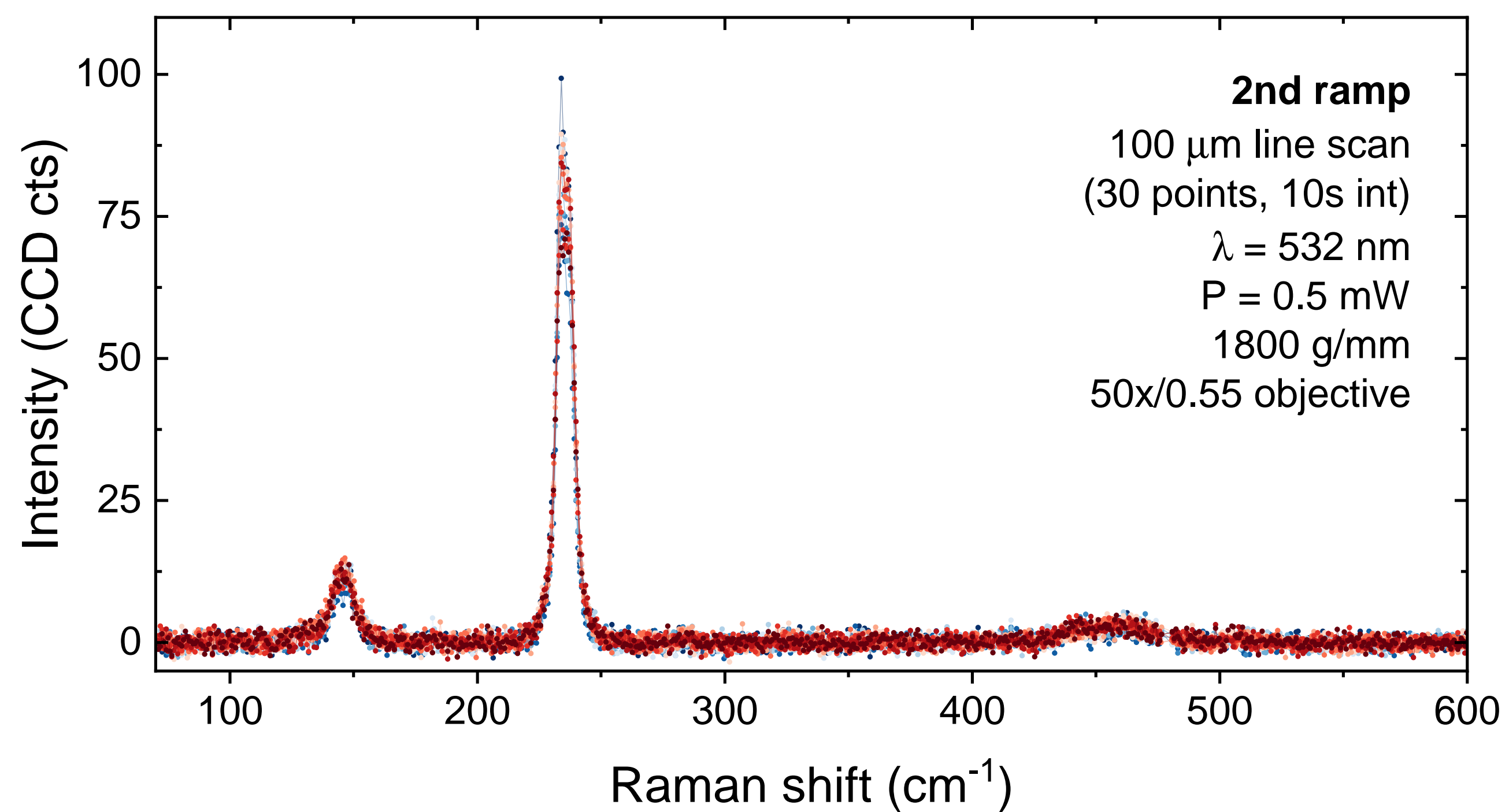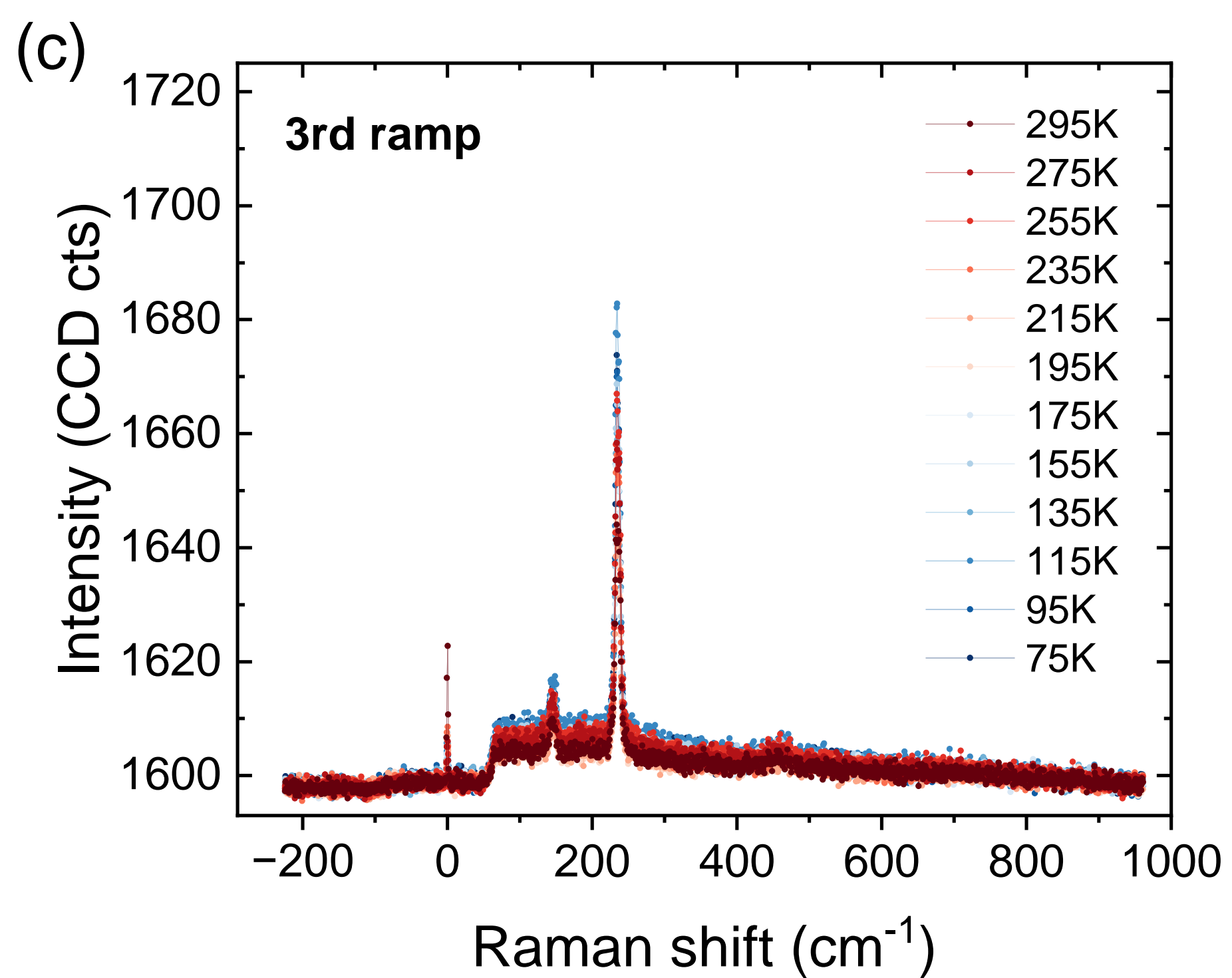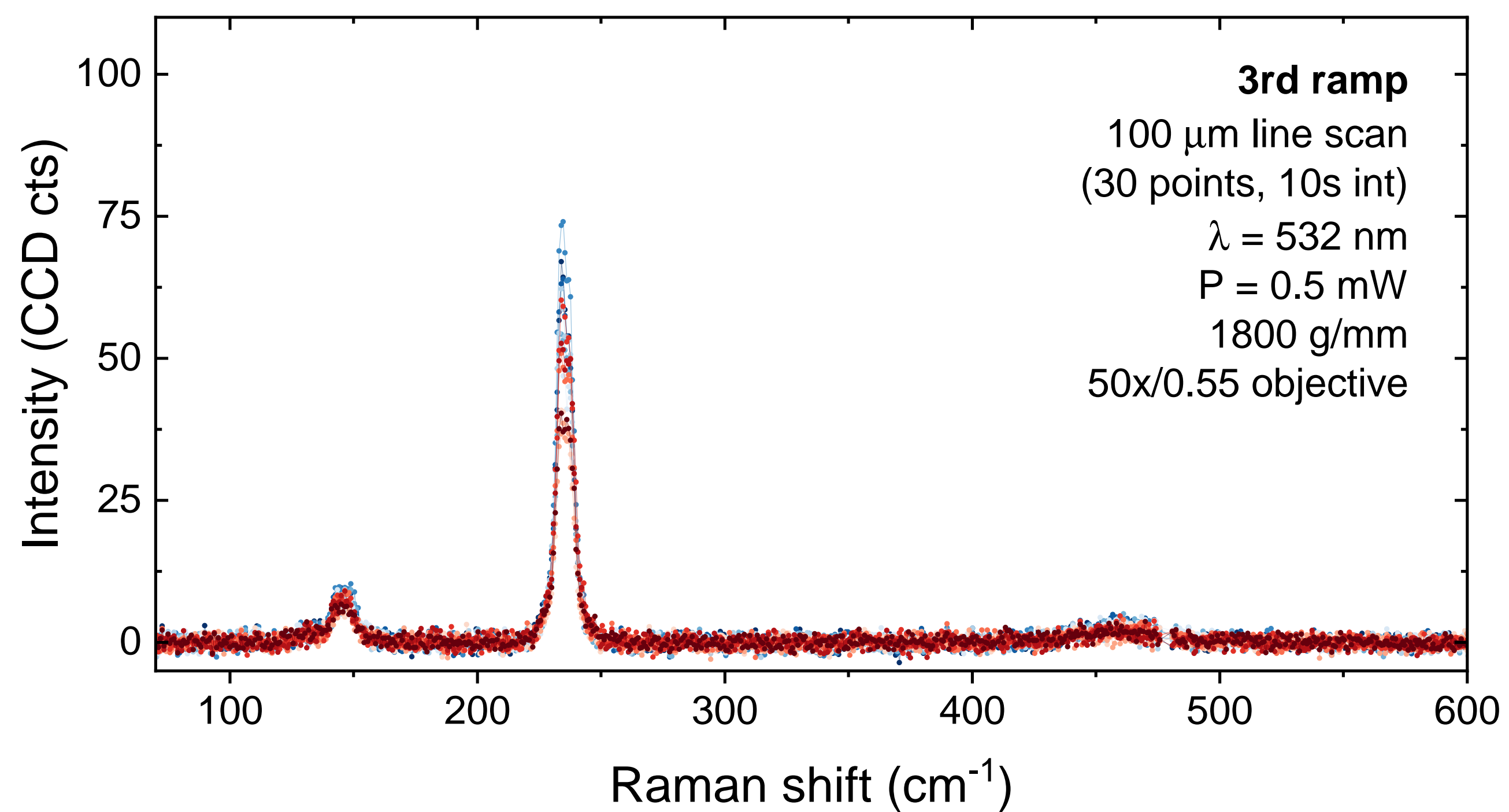

Supplement: Supplementary file 2 — Supporting File 2: smtd70463‐sup‐0002‐FigureS1‐S6.zip. [file SMTD-10-e01841-s002.zip › FigureS2.pdf]

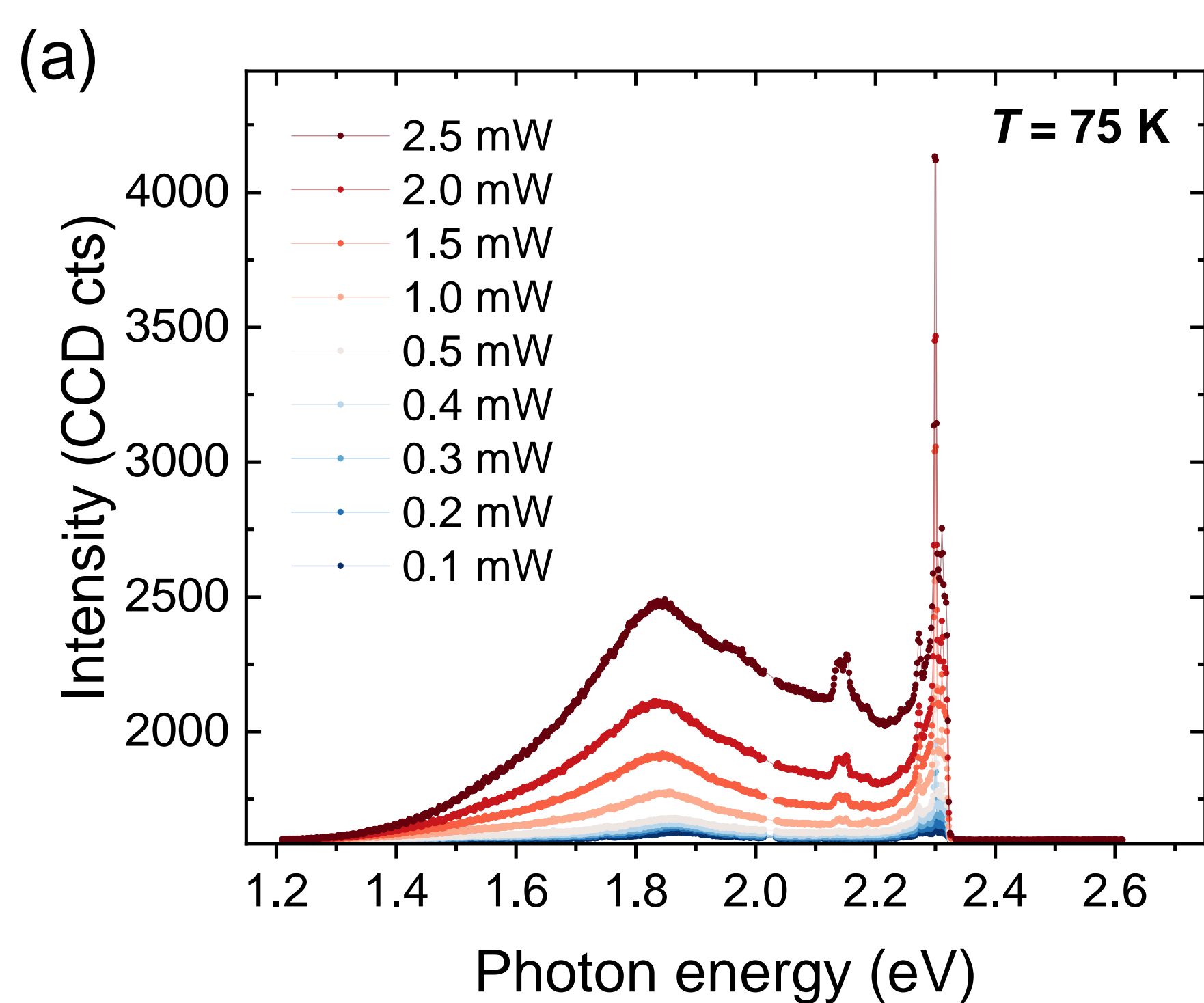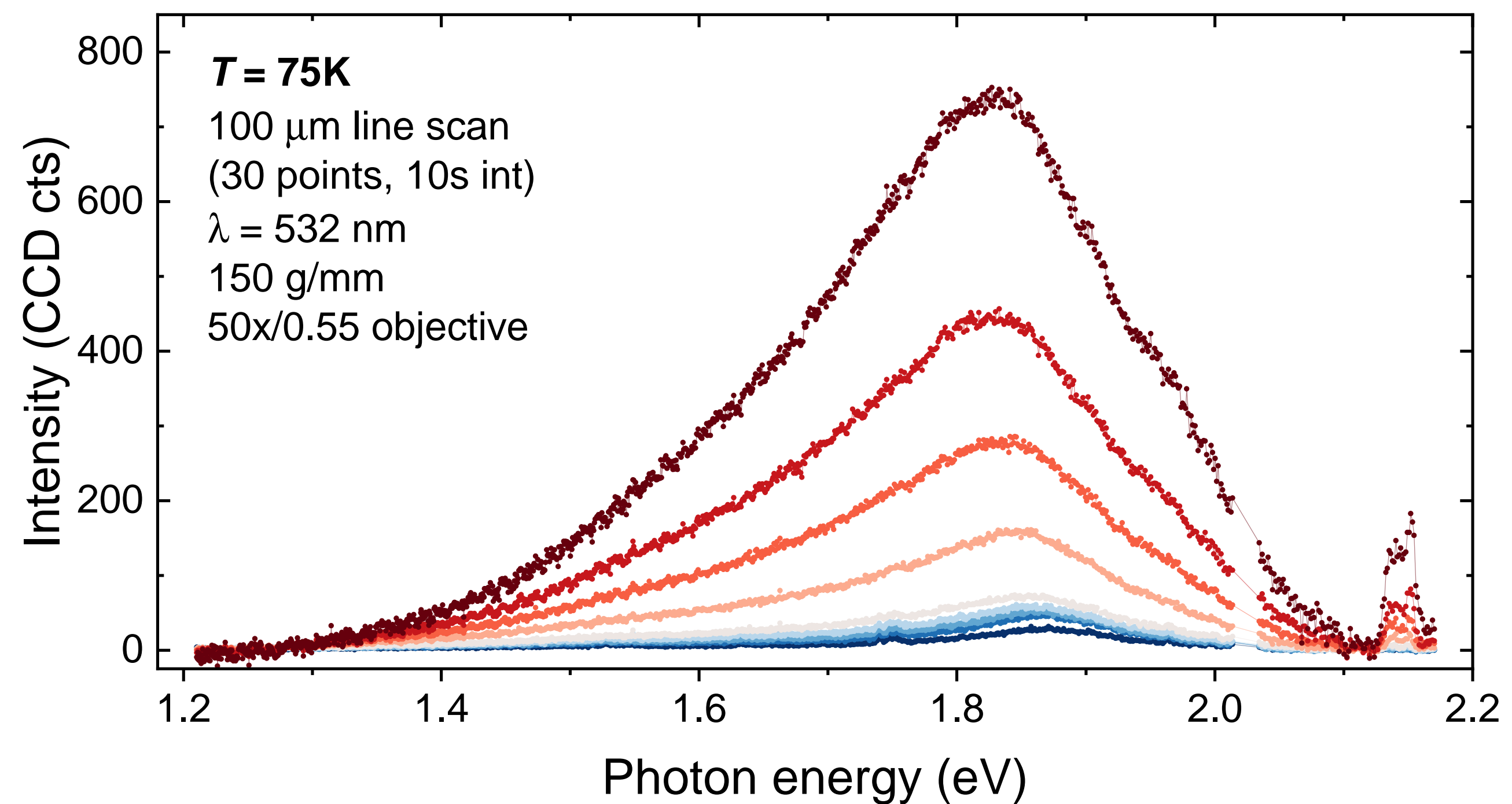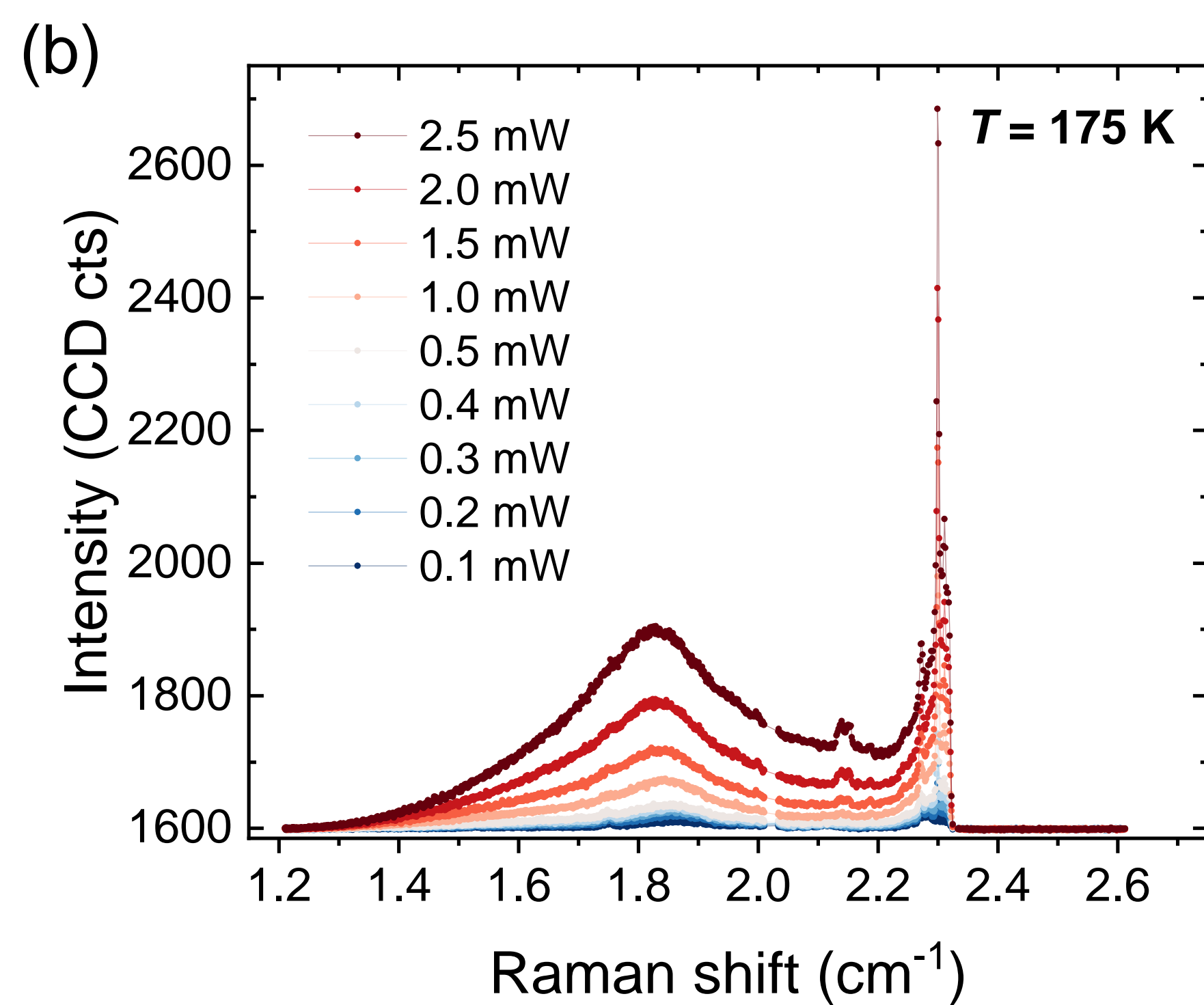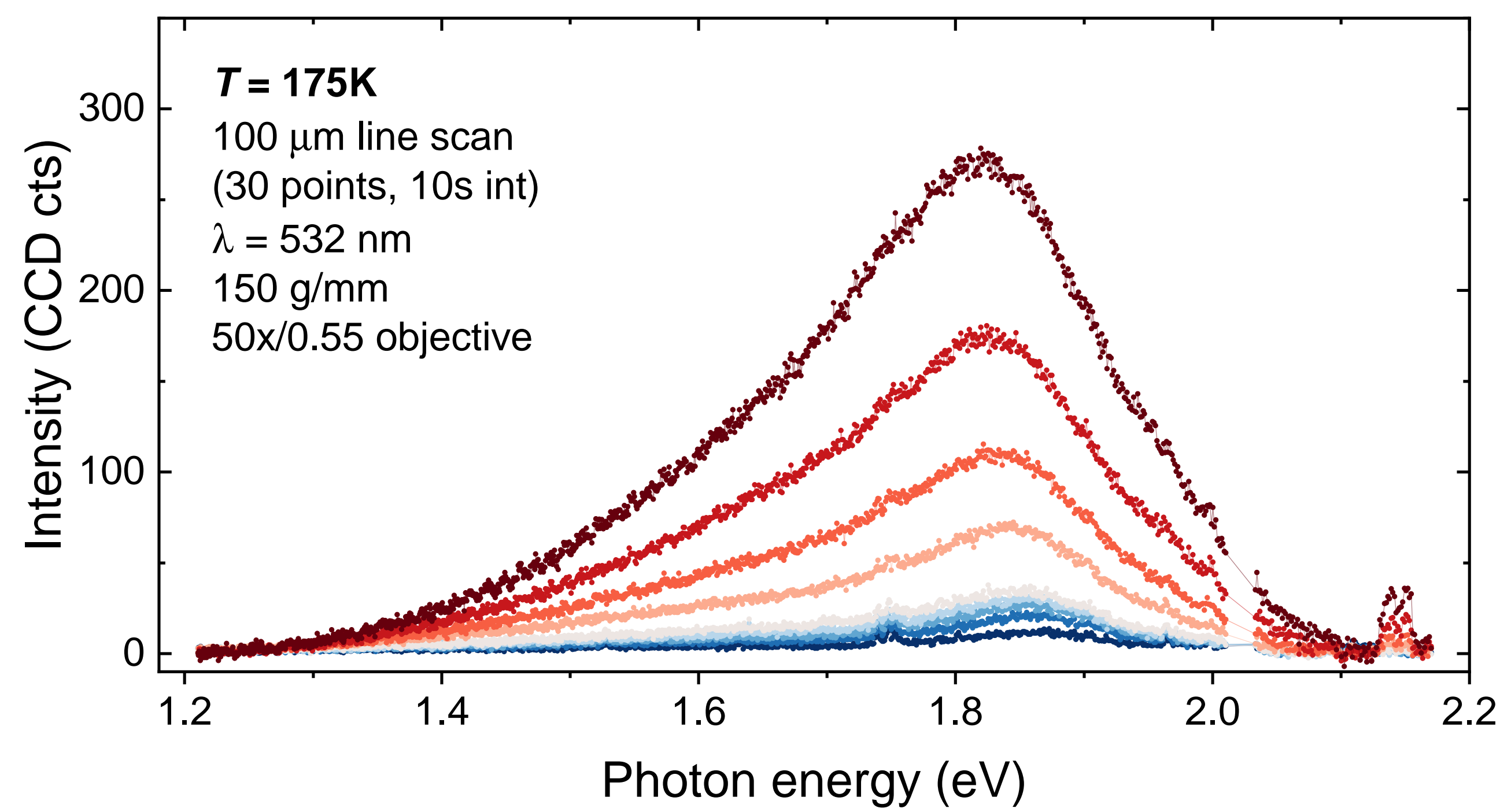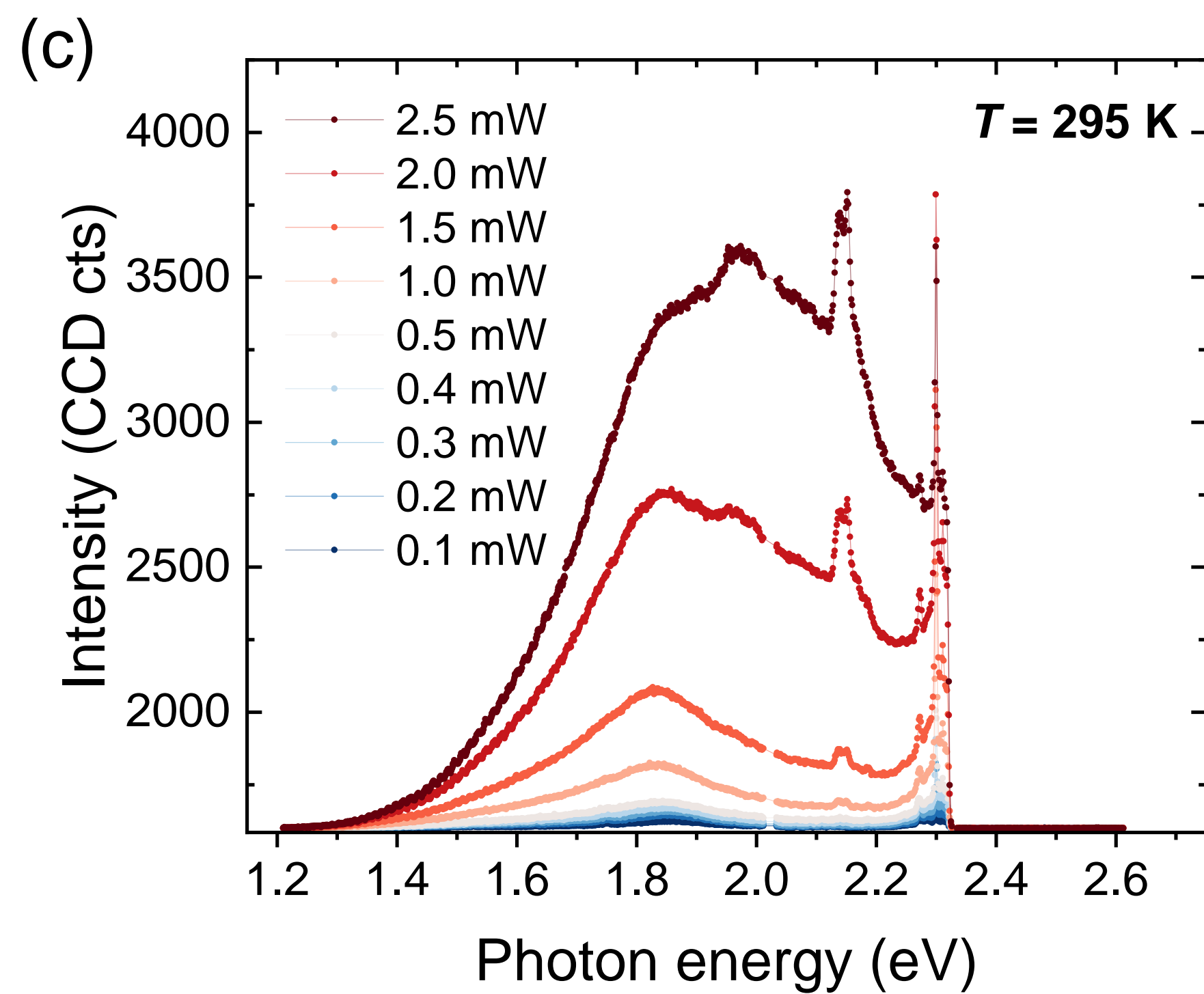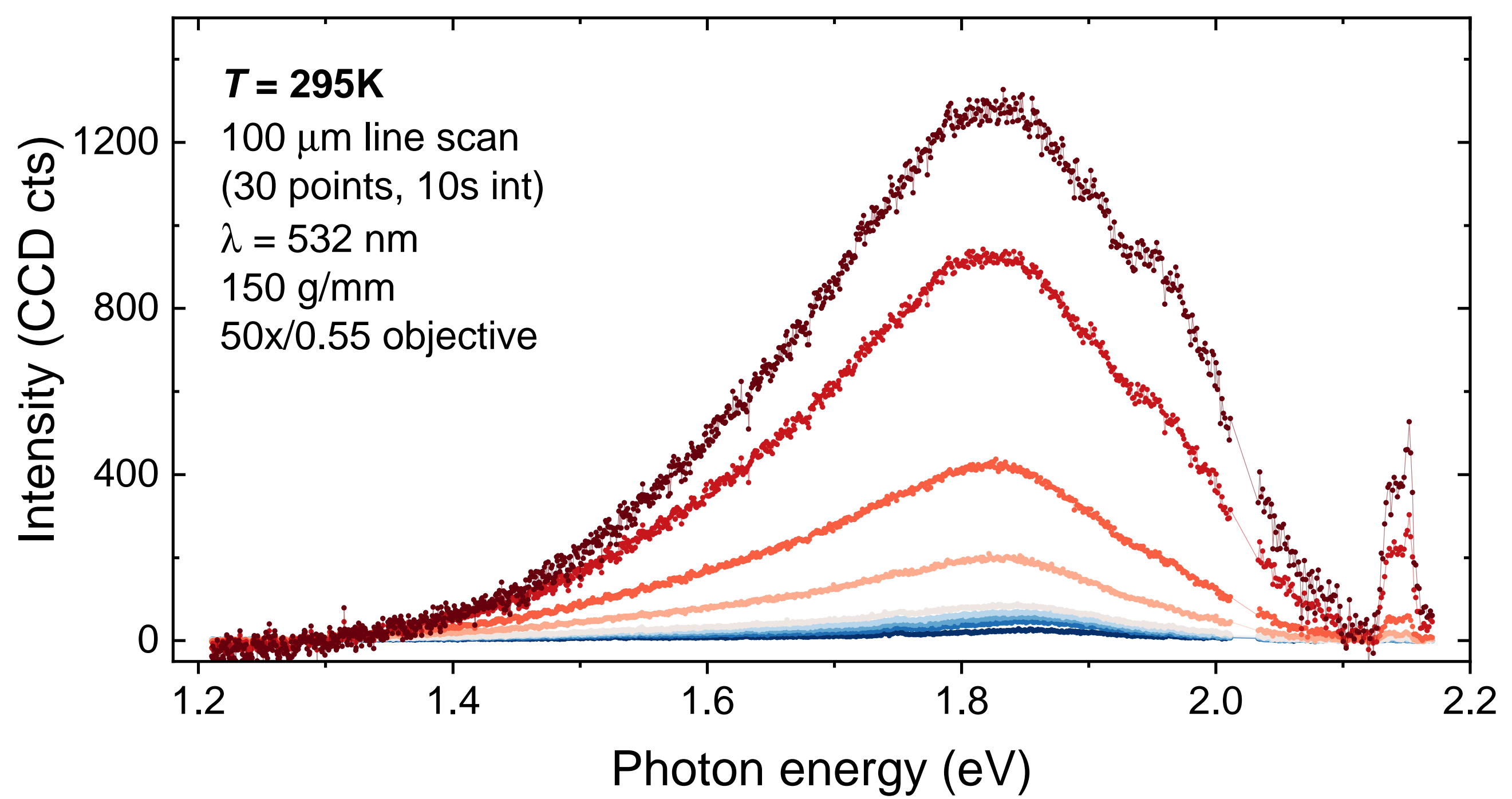

Supplement: Supplementary file 2 — Supporting File 2: smtd70463‐sup‐0002‐FigureS1‐S6.zip. [file SMTD-10-e01841-s002.zip › FigureS3.pdf]

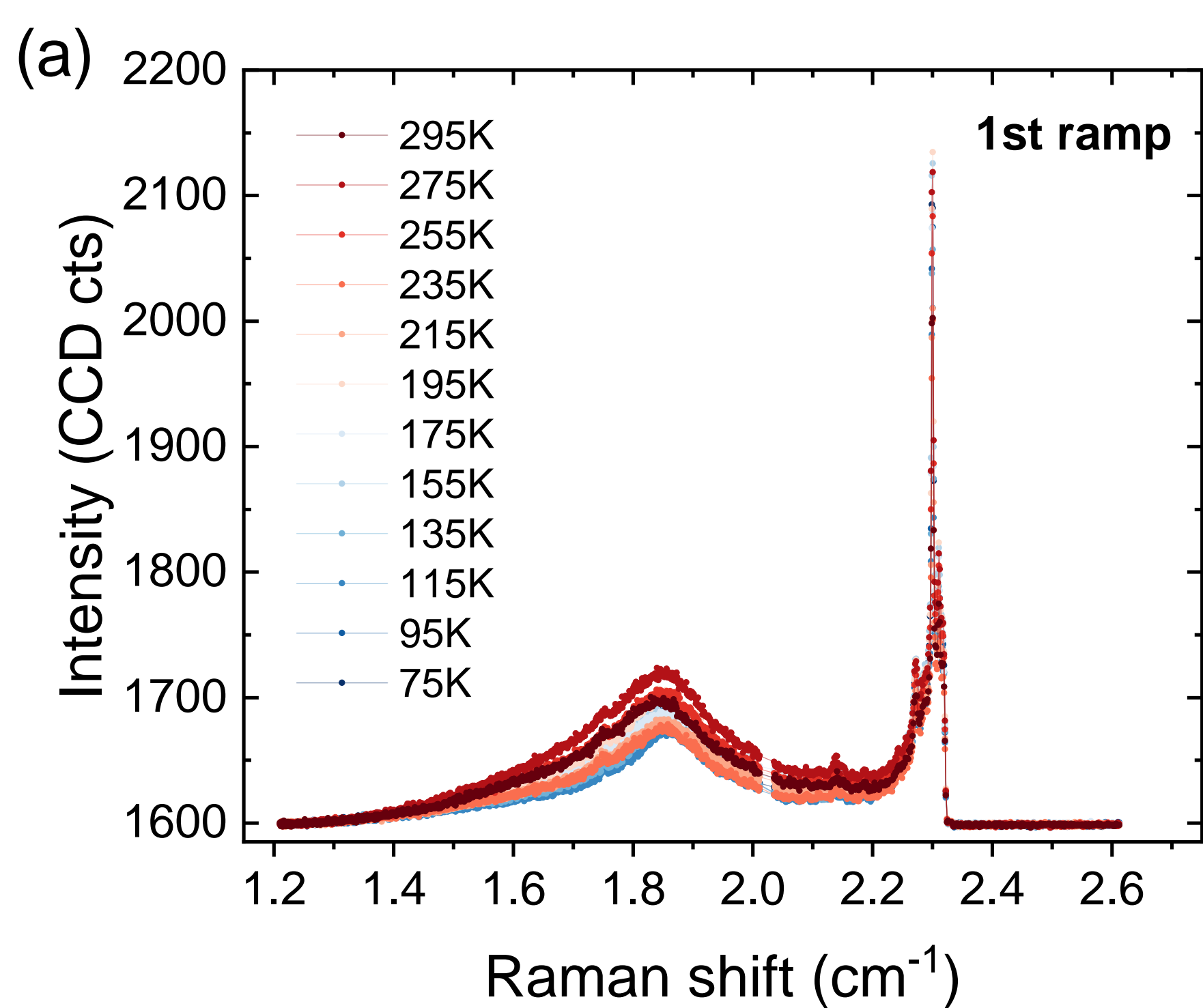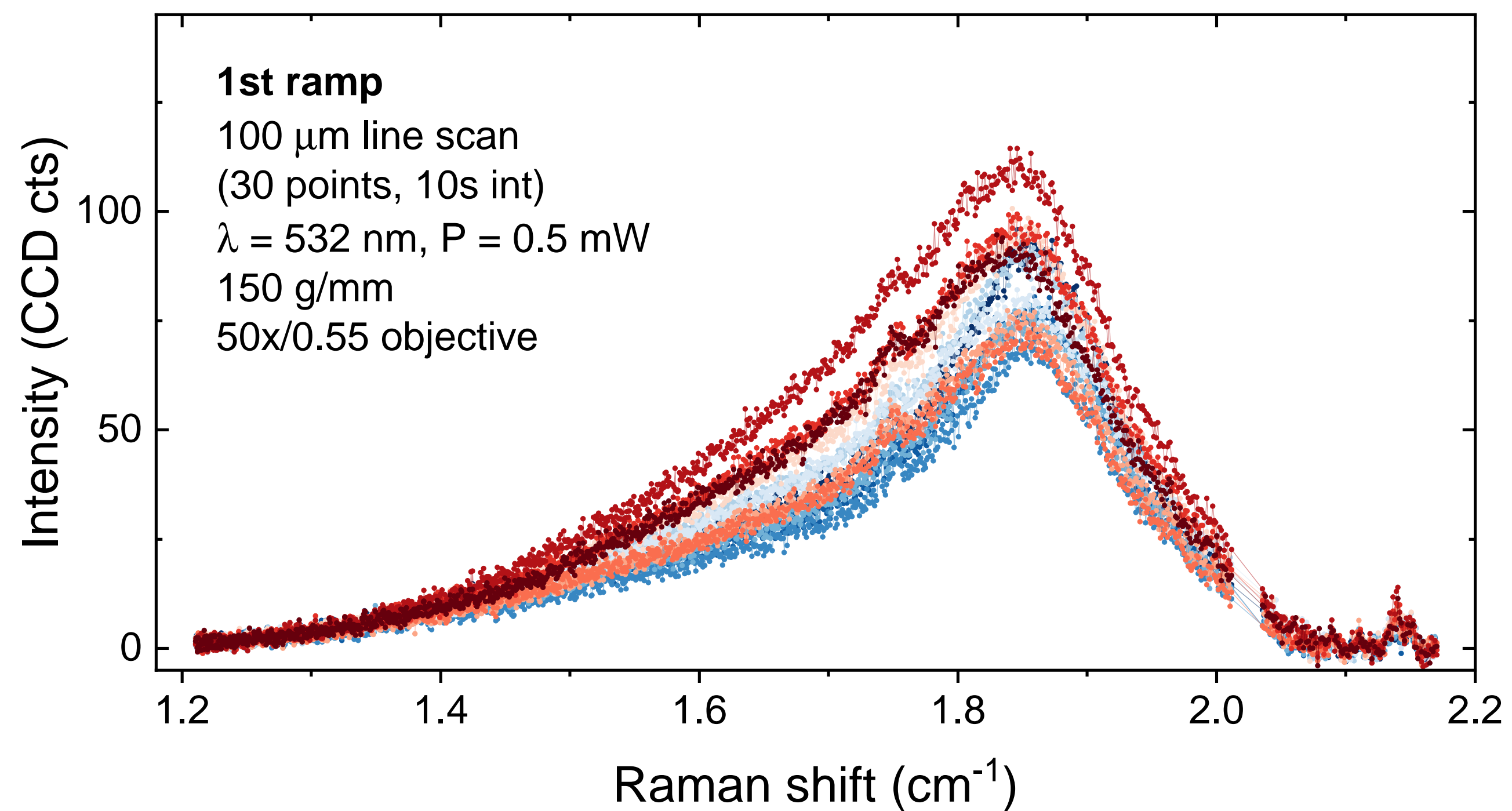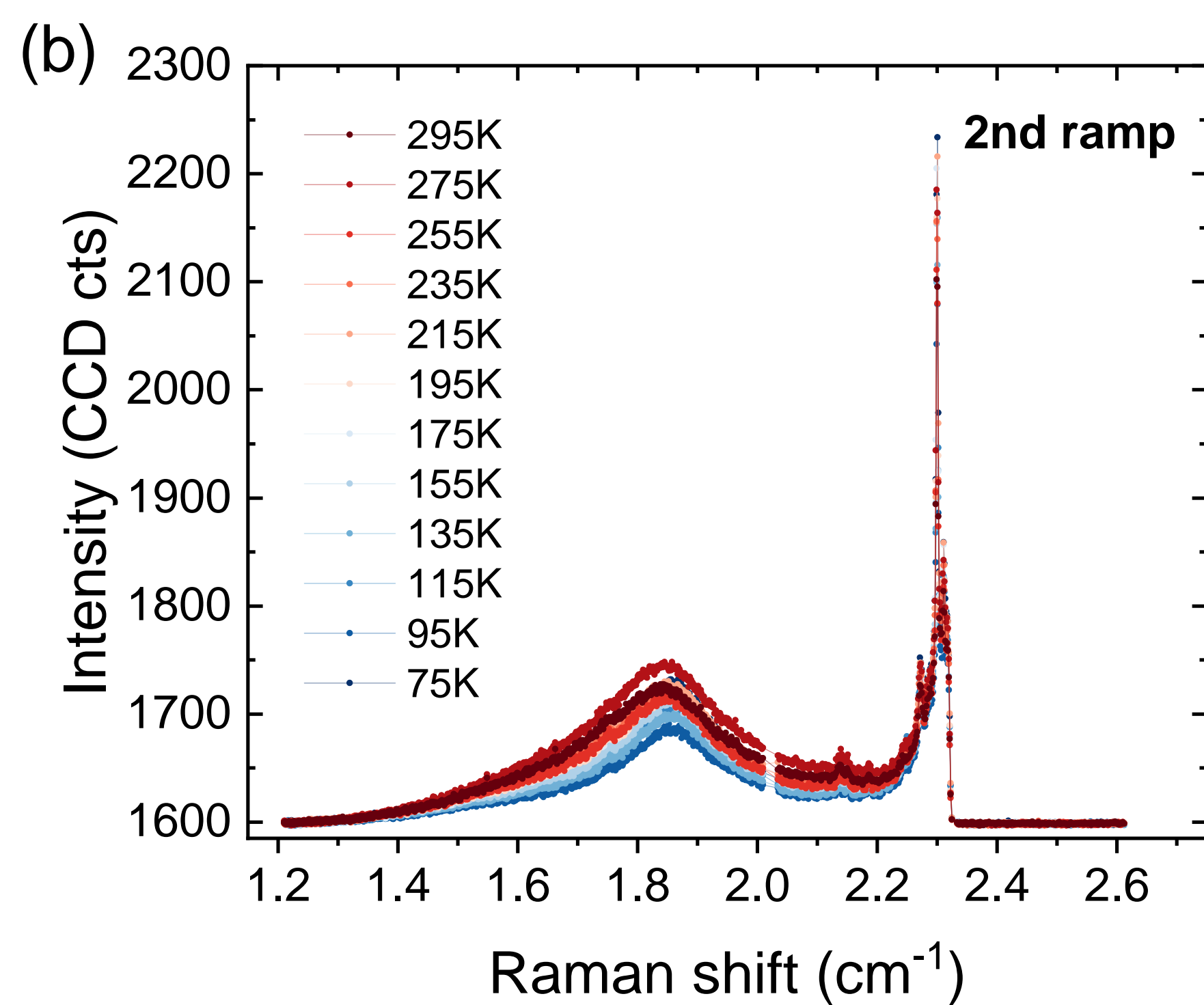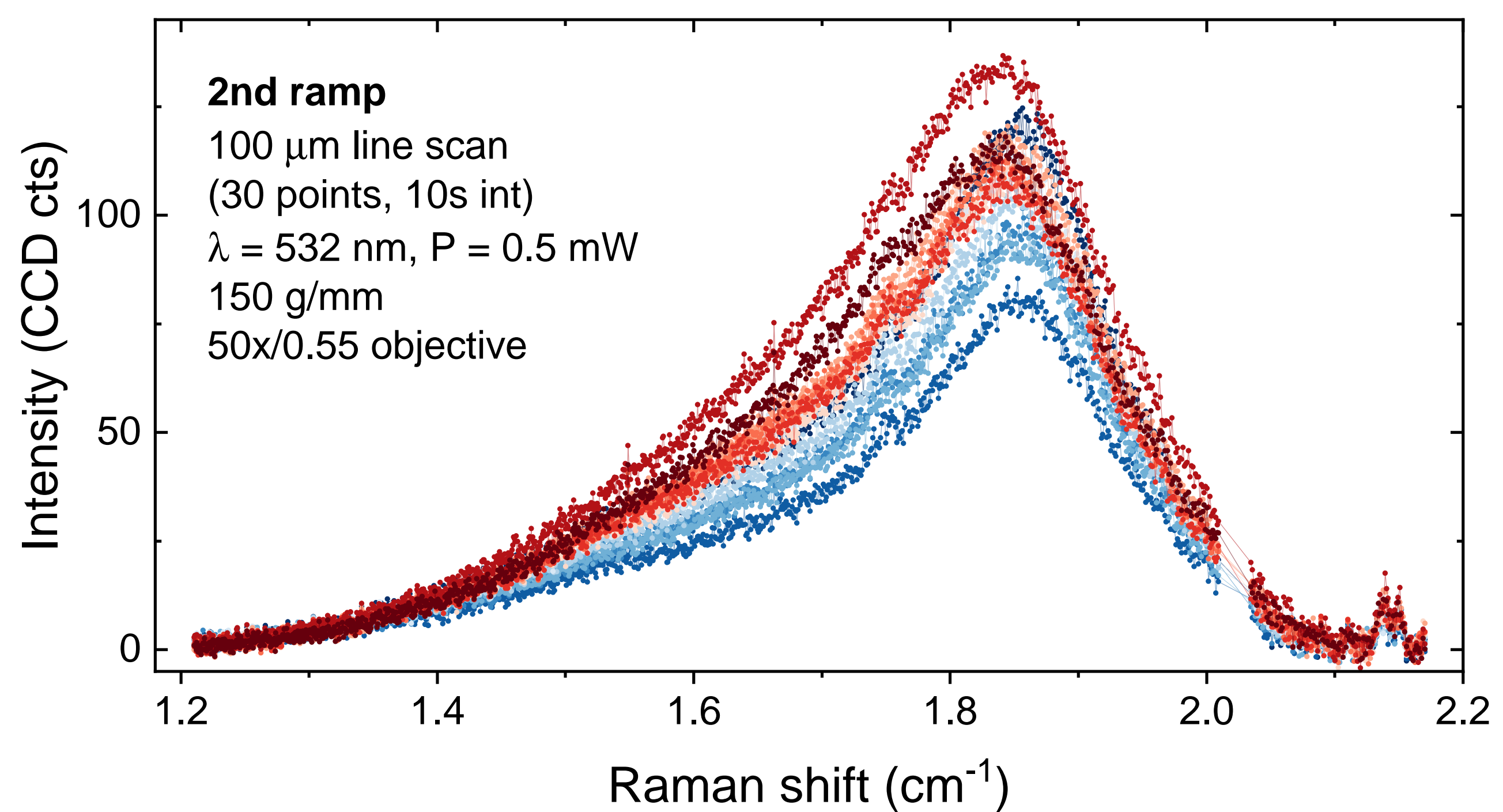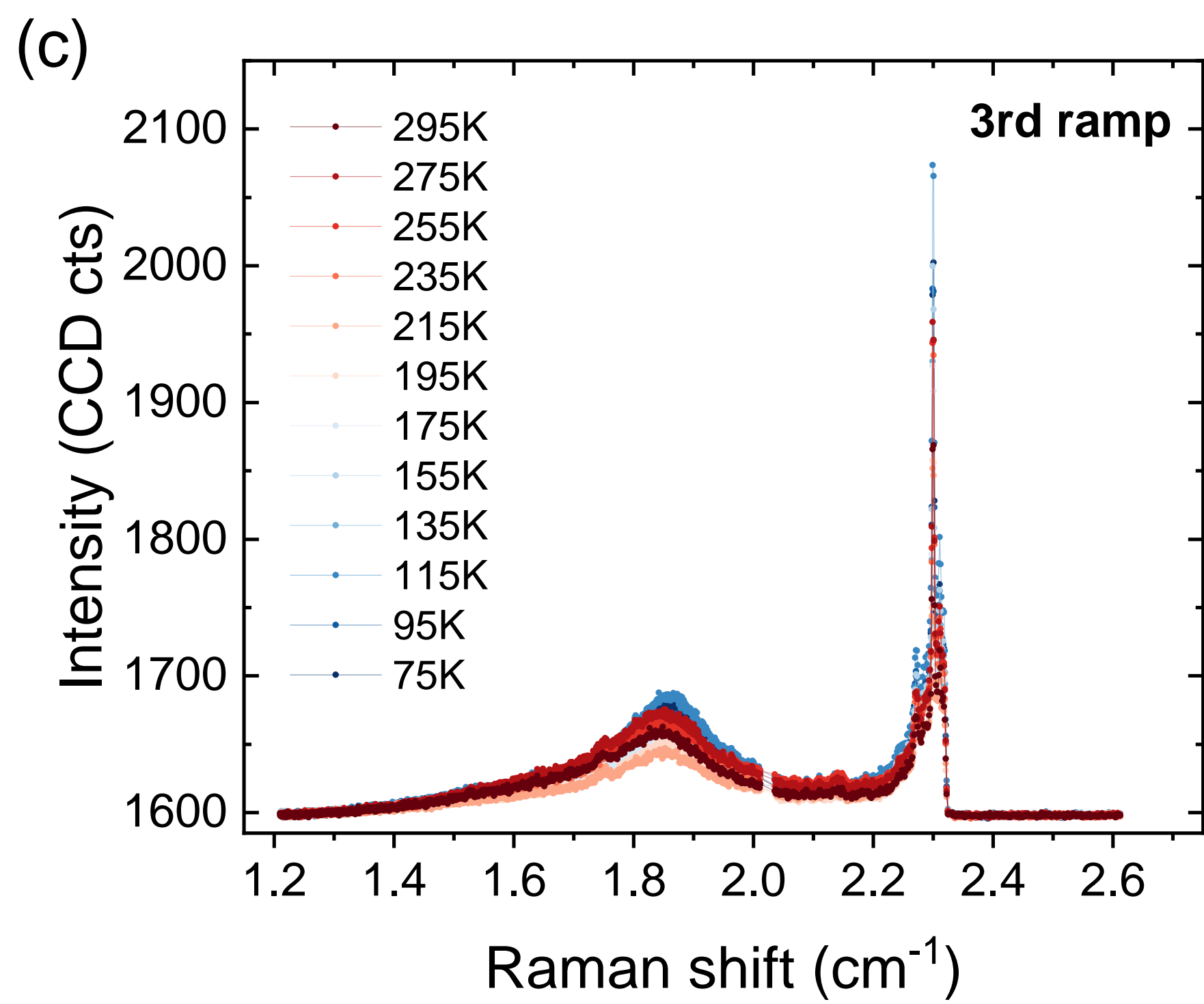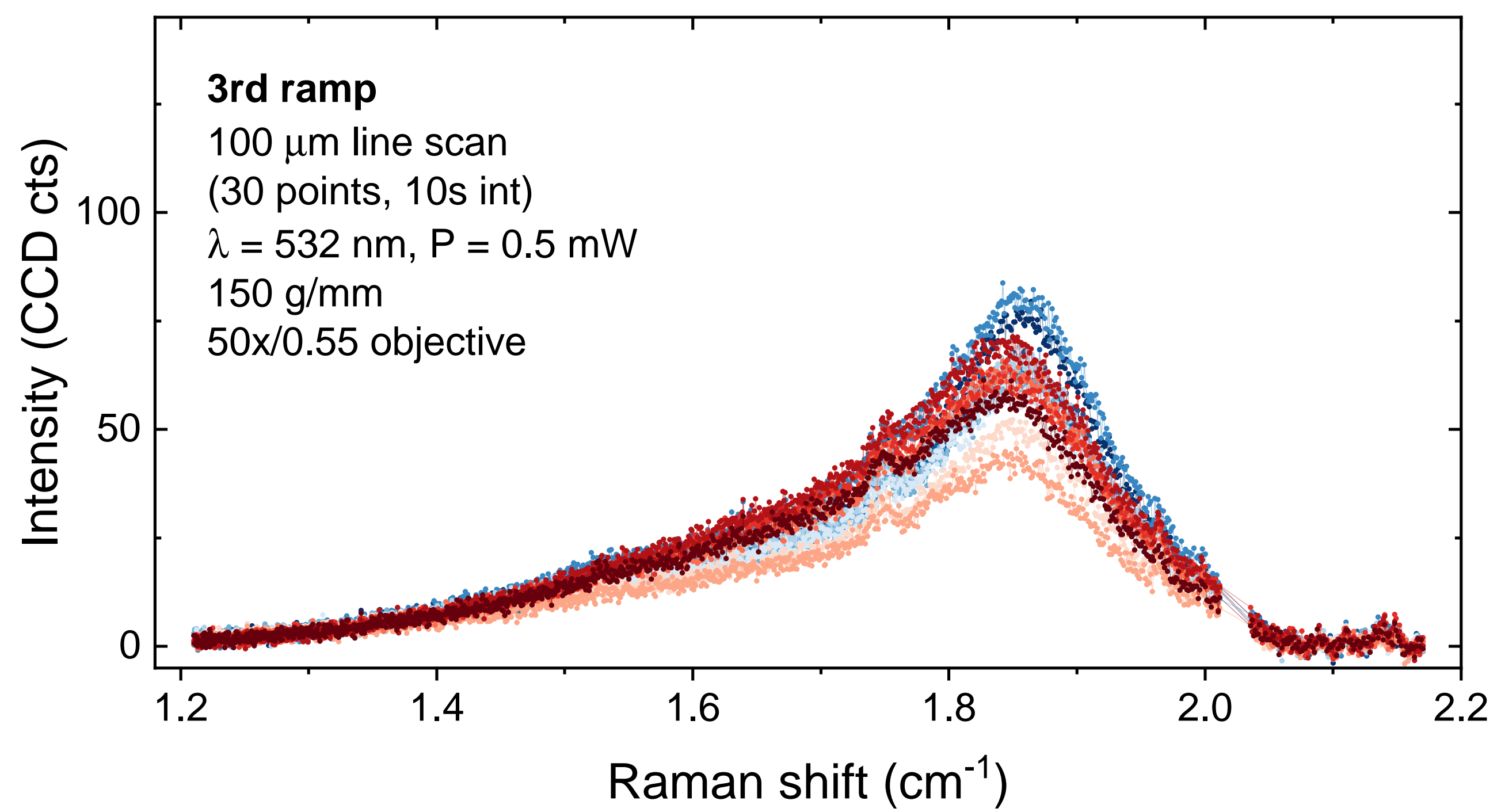

Supplement: Supplementary file 2 — Supporting File 2: smtd70463‐sup‐0002‐FigureS1‐S6.zip. [file SMTD-10-e01841-s002.zip › FigureS4.pdf]

(a)

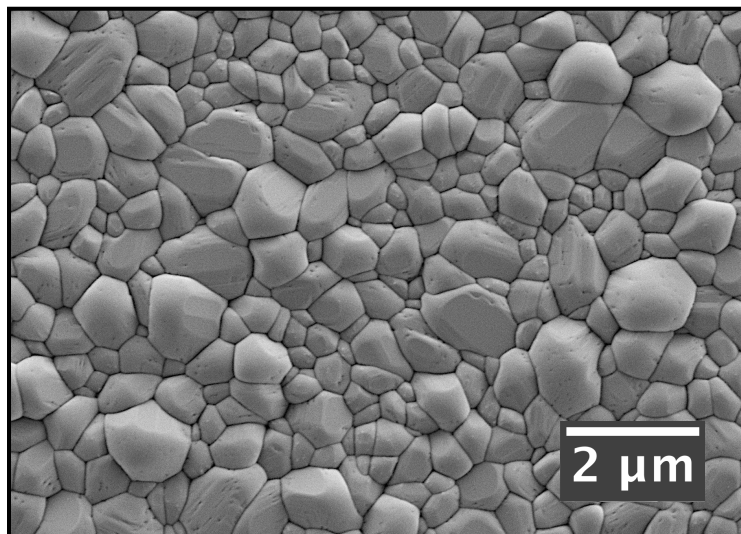

(b)

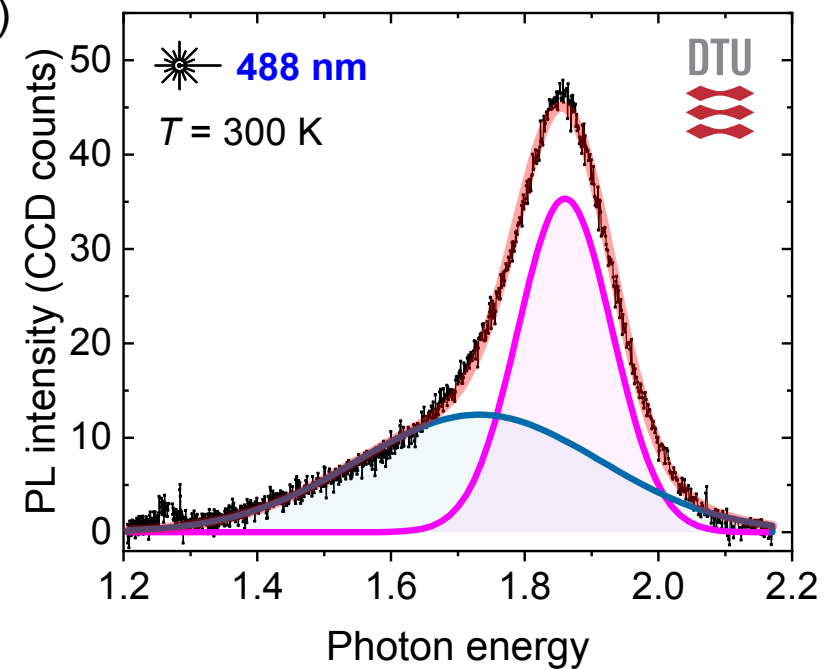

(c)

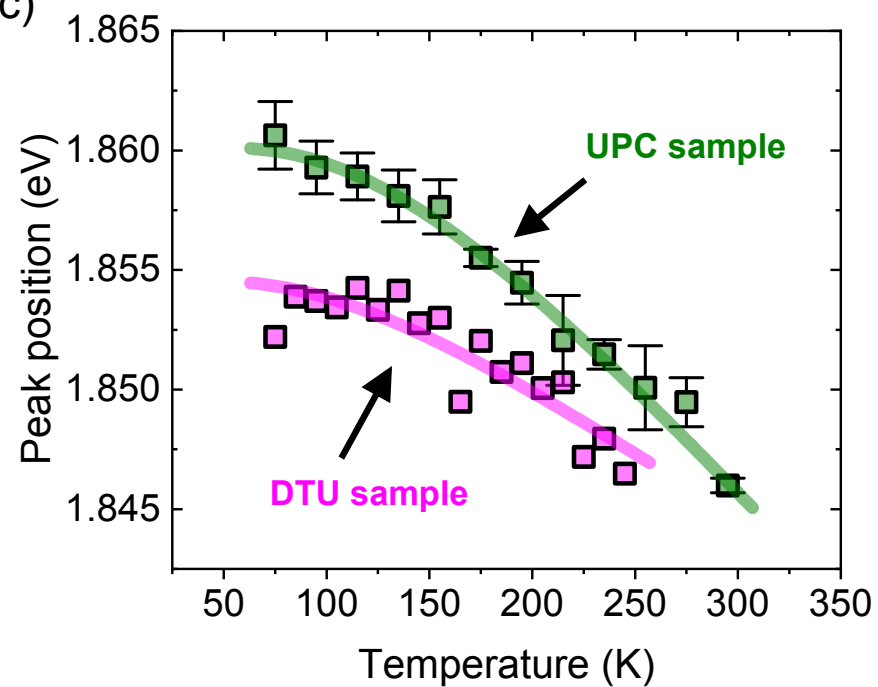

(d)

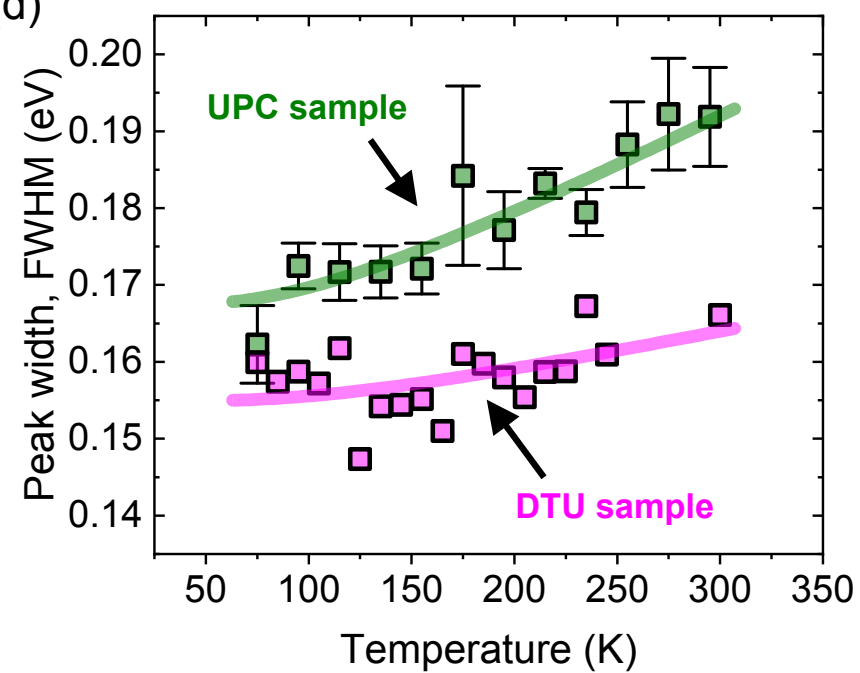

Supplement: Supplementary file 2 — Supporting File 2: smtd70463‐sup‐0002‐FigureS1‐S6.zip. [file SMTD-10-e01841-s002.zip › FigureS5.pdf]

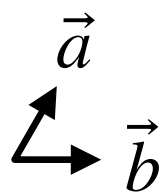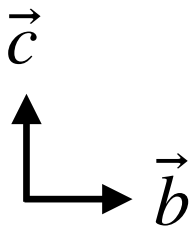

E(1)

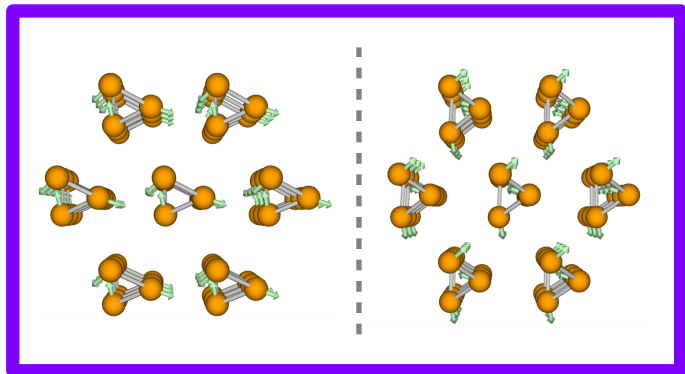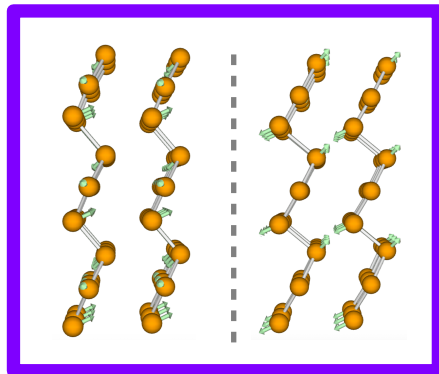

E(2)

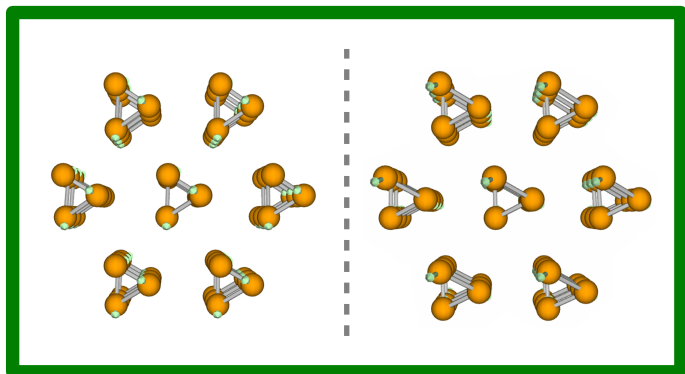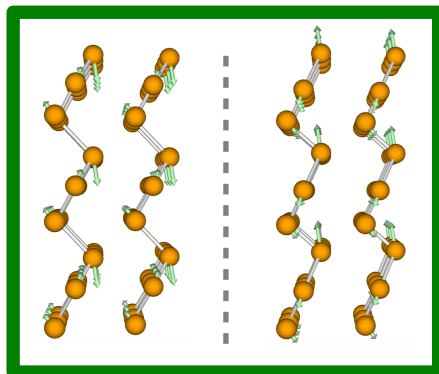

A<sub>1</sub>

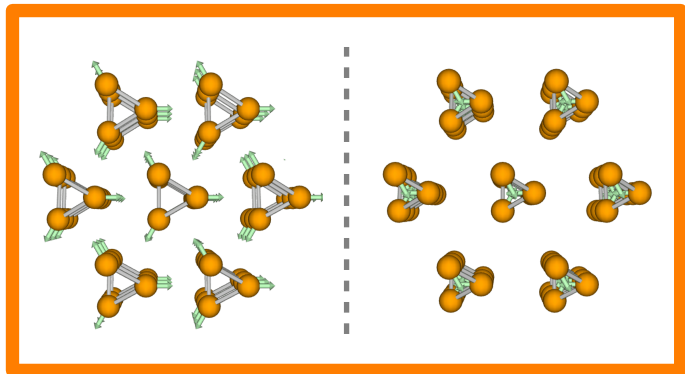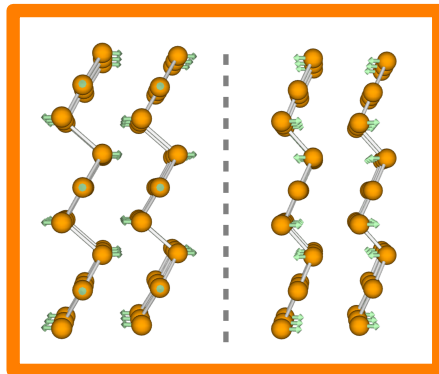

Supplement: Supplementary file 2 — Supporting File 2: smtd70463‐sup‐0002‐FigureS1‐S6.zip. [file SMTD-10-e01841-s002.zip › FigureS6.pdf]
